# Research Progress of Terahertz Technology in Microbiology

**DOI:** 10.3390/bios16090515

**Published:** 2026-09-11

**Authors:** Ding Cao, Ruibing Dong, Guangyou Fang, Xuequan Chen

**Affiliations:** 1 GBA Branch of Aerospace Information Research Institute, Chinese Academy of Sciences, Guangzhou 510700, China; caoding@aitech.edu.cn (D.C.); dongrb@aircas.ac.cn (R.D.); gyfang@mail.ie.ac.cn (G.F.); 2 Guangdong Provincial Key Laboratory of Terahertz Quantum Electromagnetics, Guangzhou 510700, China; 3 School of Photonics and Optical Engineering, Aerospace Information Technology University, Jinan 250200, China

**Keywords:** terahertz, spectroscopy, sensing, microbiology, bacteria, radiation effect

## Abstract

Microorganisms are ubiquitous in nature, and microbial activities are closely intertwined with the entire life cycle system and human life. Developing novel technologies for the detection, characterization and manipulation of microorganisms promotes their applications in clinical, environmental and industrial areas. Over the last two decades, terahertz (THz) technology has emerged as a new optical tool for microbiology. The great potential originates from the unique advantages of THz waves including the high sensitivity to water and inter-/intra-molecular motions, the non-invasive and label-free detecting scheme, and their low photon energy. THz waves have been utilized as a stimulus to alter microbial functions or as a sensing approach for quantitative measurement and qualitative differentiation. This review specifically focuses on recent research progress of THz technology applied in the field of microbiology, including two major parts of THz biological effects and the microbial detection applications. At the end of this paper, we summarize the research progress and discuss the challenges currently faced by THz technology in microbiology, along with potential solutions. We also provide a perspective on future development directions. This review aims to build a bridge between THz photonics and microbiology, promoting both fundamental research and application development in this interdisciplinary field.

## 1. Background and Introduction

The Earth is flooded with micron-sized microorganisms. Although microorganisms cannot be seen by the naked eye, they interact with human beings at every moment. For example, an adult lives with ~10^24^ microorganisms in the gut, mouth and skin [1,2], amount of which is even larger than that of human cells [3]. Microbes are not only able to cause diseases but also alter human appetite and regulate food intake by releasing proteins [4] that activate anorexic neurons in brain. Detecting microorganisms, characterizing their cellular components and investigating their microbial behaviors under various stimuli are vital for agricultural, clinical and industrial settings.

**Figure 1:**
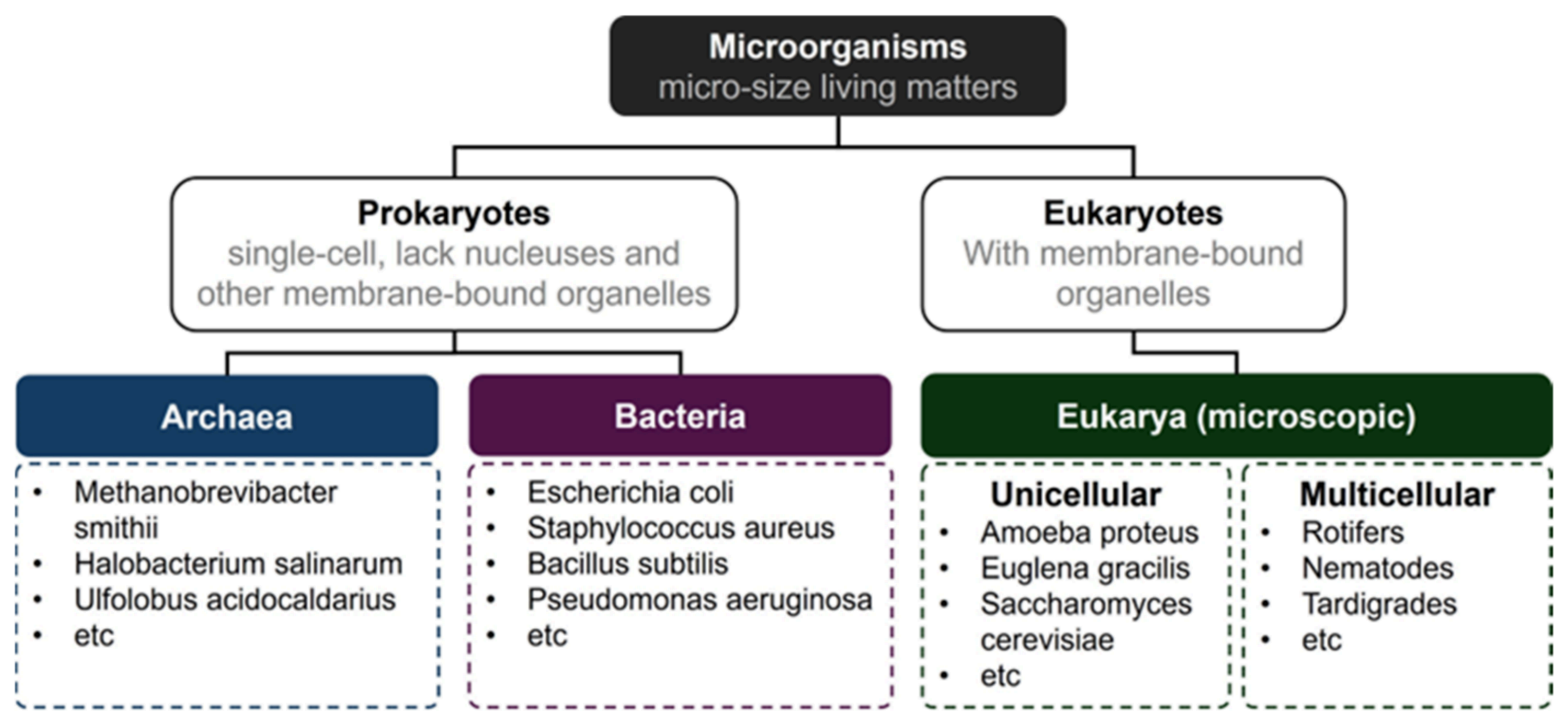
Categories and examples of microorganisms.

Microorganisms include all micro-size living things from the three-domain system of Archaea, Bacteria and Eukarya, as shown in Figure 1. Archaea and Bacteria are collectively termed as prokaryotes, defined as single-cell organisms that lack nucleuses and other membrane-bound organelles. Eukarya, in contrast, comprises membrane-bound organelles. Animals and plants are also eukaryotes; however, only unicellular and microscopic multicellular Eukarya such as *amoeba proteus* and *rotifers* are considered microorganisms. Both unicellular form (microns in length) and colony form (larger than centimeter) are common morphologies of microorganisms in daily life. In addition, although viruses are not generally considered as living things and are not classified in the three-domain system, THz virus research adopts similar protocols to those used for living microorganisms considering their comparable size. Therefore, we will consider viruses as microorganisms in this review.

Although there have been numerous technologies for studying microorganisms, as shown in Table 1, there are still many yet-unknown properties and application potential, highlighting the continued need for novel research tools. In recent decades, a less-explored electromagnetic spectrum, the THz regime (10^11^ Hz to 10^13^ Hz), has become an emerging research area. Scientists are curious about how THz waves interact with things (especially bio-materials), since THz radiation is missing from natural sunlight due to the strong atmosphere absorption (mainly water vapor [5]). The interactions of microorganisms with other spectral radiation are better understood. For example, infrared light can enhance cell proliferation [6] whilst ultraviolet light can destroy cellular structures [7]. Studies on the interaction between THz radiation and microorganisms may reveal some unseen biological effects during cellular growth.

**Figure 2:**
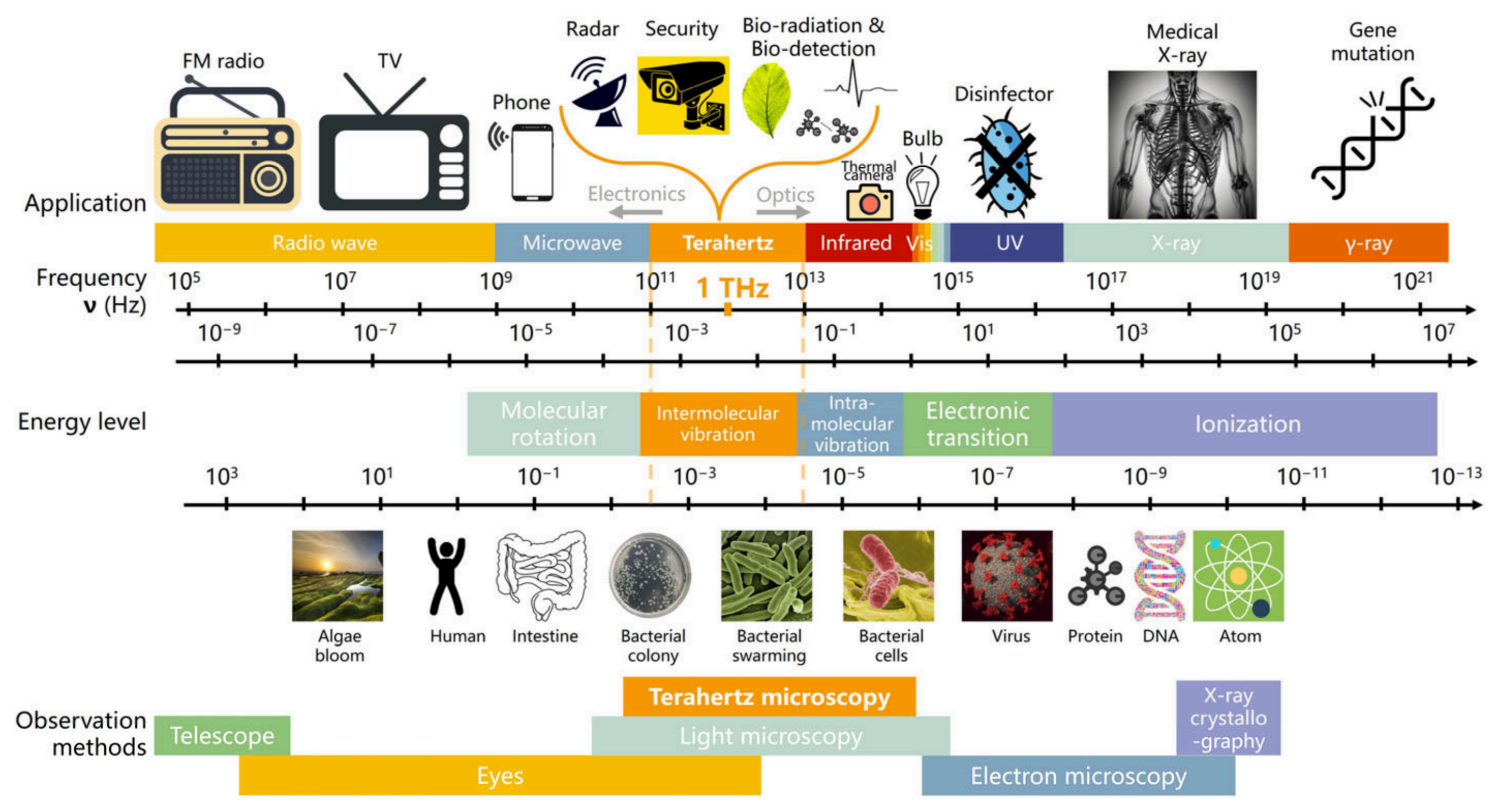
Comparison of electromagnetic wave spectrum and size of microorganisms and their sub-cellular components. Images are from pixabay.com (accessed on 15 Mar 2026) with permission.

THz waves are located in between microwaves and infrared, as shown in Figure 2. Throughout the electromagnetic spectrum, each frequency range has found unique applications, such as wireless communications by microwaves and radio waves, visual applications by visible light and medical diagnosis by X-ray. In contrast, THz waves are less studied due to the historical lack of efficient sources and sensitive detectors. With advancing instrumentations, THz photonic technology has become more widespread nowadays [8], especially in biomedicine [9]. THz waves have similar energy levels to intermolecular motions of biomolecules, making THz spectroscopy sensitive to characteristic phonon absorption of biomaterials [10]. THz frequencies also correspond to hydrogen-bonding relaxations [11]; hence, water presents strong dispersion and absorption in the THz range, highly contrasting those of biological macromolecules. The interaction with hydrogen-bonding networks also makes THz waves a sensitive probe for the hydration shell around biomolecules [12,13,14]. Strong THz fields can even modify the hydration status [15], which may affect subsequent molecular dynamics.

Although there have been a few THz biomedical reviews focused on mammalian cells and tissues [9,16,17], microorganisms have rarely been comprehensively compared, summarized, and discussed. This review aims to fill the gap in this area by conducting a systematic investigation, organization, analysis, and outlook on THz-related research in microbiology. Our goal is to provide valuable insights for researchers in related areas of THz photonics, microbial biology, and biophysics. This paper is organized as follows. In Section 2, we will summarize findings about the biological effects of microorganisms under THz radiation, taking THz waves as stimulus to investigate how microorganisms respond to the radiation. Section 3 will discuss detecting technologies developed for microorganisms based on THz spectroscopy. Finally, we discuss the current challenges and envision future outlooks in the last section.

## 2. Biological Effects on Microorganisms Under Exposure to THz Radiation

THz technology has seen rapid progress in biomedical applications. The development of high-power THz sources is one of the driving forces. Nowadays, intense THz radiation can be generated through various approaches, including solid-state devices (e.g., Guun diodes and Schottky diodes) [18], spintronic devices [19], non-linear crystals [20], air plasma [21], quantum cascade lasers (QCLs) [22] and free electron lasers (FELs) [23]. Even fiber-coupled photoconductive antennas can reach milliwatt-level output power [24]. The effects of THz radiation on humans and biological organisms have attracted increasing scientific interest for several reasons. First, THz radiation has rarely been involved in the biological evolution process because of the strong absorption by the atmosphere. How living organisms respond to THz waves remains an open question. It is known that the high-water concentration of tissues significantly attenuates the THz light to a penetration depth of tens to hundreds of micrometers [25], hence the radiation influence on animals is limited to the upper skin layers. However, such thickness is already beyond the size of a microorganism and could completely change its behavior. Second, curiosity stems from the unique properties of the THz wave. Specifically, the frequency of THz waves matches well with the intramolecular motions of DNA and proteins. More importantly, as illustrated in Figure 2, the energy level of these waves corresponds to the hydrogen-bonding relaxations and van der Waals force, both of which play important roles in intermolecular interactions. Owing to these interactions, THz waves can induce non-thermal biological effects by altering intermolecular interactions without breaking molecular bonds, positioning them as promising tools for modifying the configuration of biological molecules and the physiological state of living cells. Individual studies have reported diverse non-thermal radiation effects on different biological samples [26,27,28,29]. Recent reviews have summarized these findings and highlighted the complexity of these effects [30,31,32], which can be influenced by a wide range of parameters, including physical irradiation conditions (e.g., frequency, power, and peak electric-field strength) and biological factors (e.g., molecular or cellular targets, tissue type, and culture conditions).

In 2015, the Scientific Committee on Emerging and Newly Identified Health Risks (SCENIHR), guided by the European Commission, shared its detailed opinion on the potential health effects of exposure to electromagnetic fields [33]. This important document highlights that, as THz technology advances, there is a growing need to study the potential biological effects of THz exposure. SCENIHR specifically recommended more research on the effects of THz radiation on the skin (for long-term and low-level exposure) and on the cornea (for short-term and high-intensity exposure). Over the past decades, researchers have investigated the effects of THz radiation on skin cells [34,35], nerve cells [27,36], corneal tissues [37,38] and so on. For example, some studies found increased gene expression and cell proliferation in skin cells after THz exposure [39,40,41,42], whilst some studies suggest no radiation effect [43,44,45]. Changes in gene expression [46], cell membrane properties [47], and overall cell viability [48] have also been reported on nerve cells. For example, a study by Zhao et al. [46] exposed mice cortical neurons to THz radiation from a QCL source (70 μW/cm^2^ @ 3.1 THz) and found increased neurite outgrowth and synaptic-associated gene expressions, leading to increased excitatory synaptic transmission and neuronal firing. It should be noted that the radiation conditions for different works could vary significantly, as summarized in Table 2.

In comparison to mammalian cells, microorganisms typically have higher cell count [1,2], exhibit greater cellular diversity [49], and possess a simpler cellular structure [50]. Their existence can manifest as single-celled entities or as complex colony communities, with their form often influenced by species type, growth phase and environmental conditions [51,52,53]. Notably, a single-cell microorganism contains a complete set of genetic materials, enabling it to survive and reproduce independently. In contrast, mammalian cells operate in a more interconnected manner, described metaphorically as ‘cogs in a machine’, working in unison to create functional organs. Many microorganisms are easy to culture and exhibit strong viability and practicability in agricultural [54], industrial [55] and clinical settings [56]. Furthermore, many studies about biological effects on mammalian organisms are restricted to the outermost layer (hundreds of micrometers) due to the limited penetration depth of THz waves in highly hydrated layers. There are also difficulties in establishing large-area uniform exposure and suitable evaluation biological parameters. These limitations make it challenging to study the comprehensive effects of THz radiation on entire tissues, organs, or living organisms. In contrast, microorganisms can be fully penetrated by THz waves, which may lead to altered gene or protein expression levels [57,58,59,60]. Despite their significance, our understanding of the interactions between microorganisms and THz radiation remains limited, especially when compared to the knowledge amassed on mammalian cells. This research gap highlights the need for more targeted research in this area. In the rest of this section, we will review existing literature on the biological effects of THz radiation on microorganisms, compare these findings with those on mammalian cells and discuss the potential future directions this research might take. In order to maintain the fluency of the article and facilitate better cross-comparison, the diverse experimental conditions, including THz sources and types, radiation frequencies and intensity, as well as exposure time and experimental temperature, are summarized in Table 2 rather than elaborated upon in the main text.

### 2.1. THz Radiation Effects on Microorganisms

**Figure 3:**
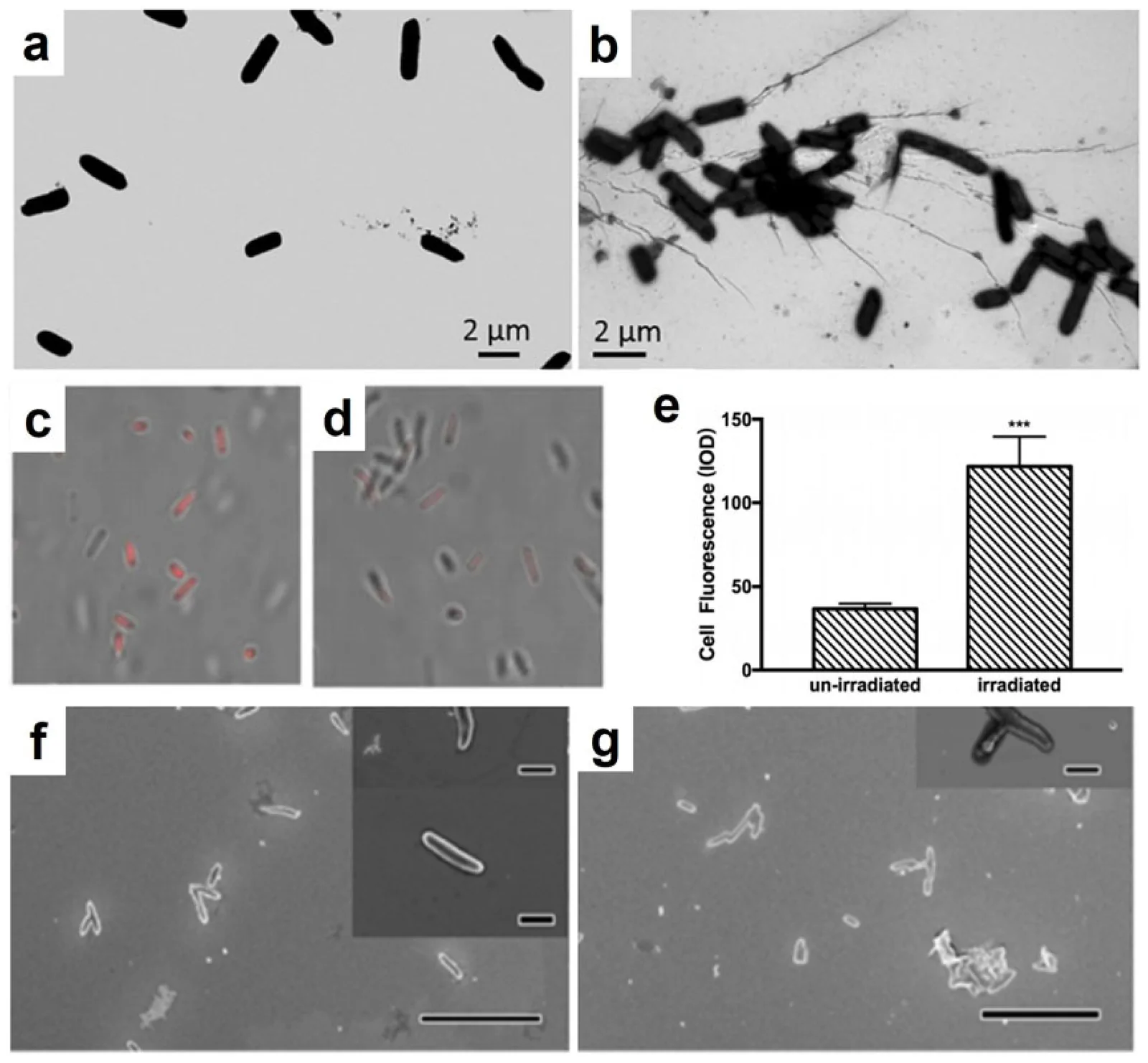
(**a**) Stand-alone bacteria before irradiation and (**b**) aggregated bacteria after the irradiation. Panels (**a**,**b**) are reproduced with permission from Peltek et al., Sci. Rep. 11, 20464 (2021) [62]. Copyright 2021 the authors, licensed under a Creative Commons Attribution (CC BY) license. (**c**) Merged optical images and fluorescent images of *E. coli* cells after exposure to THz radiation and (**d**) the untreated control. (**e**) Quantification of fluorescence intensity of *E. coli* cells after exposure to THz radiation. *** in the figure refers to *p* < 0.001. Panels (**c**–**e**) are reproduced with permission from Zhao et al., Biomed. Opt. Express 11, 3890 (2020) [63]. Copyright 2020 Optical Society of America. SEM images showing that (**f**) following 10 min of THz exposure from a synchrotron source, *E. coli* cells exhibited a dehydrated appearance, accompanied by cytosolic leakages. (**g**) At 90 min of THz exposure, *E. coli* cells had altered morphology. Scale bars in SEM images are 10 μm, and inset scale bars are 1 μm. Panels (**f**,**g**) are reproduced with permission from Ivanova et al., ACS omega 9, 49878 (2024) [65]. Copyright 2024 the authors, licensed under a Creative Commons Attribution-NonCommercial-NoDerivatives (CC BY-NC ND) license.

Exposure to THz radiation has been observed to stimulate gene and protein expression and enhance cell metabolism in mammalian cells [40,42,48,61]. For microorganisms, *Escherichia coli* (*E. coli*) has been mostly studied to understand the biological effects of THz radiation. Numerous findings indicated that THz radiation can affect bacterial metabolism. For example, in a series of experiments by Peltek et al. [62], they revealed an upregulation in genes associated with cell aggregation and adhesion after exposure to 2.3 THz radiation with an intensity of 1400 mW/cm^2^ from an FEL source (Figure 3a,b), while those related to cell division showed decreased activity. In another study [63], *E. coli* were exposed to a 3.1 THz CW for an 8 h duration, and an increment of the plasmid copy number was observed, which subsequently led to a rise in the production of red fluorescence protein (Figure 3c–e). Shifting the focus to the transcriptional level, Imashimizu et al. [64] centered their research on the RNA polymerase activity of *E. coli* under 4.0 ± 1.0 THz FEL radiation. Their results indicated significant impact in transcription processes, including abortive initiation and pausing. Focusing on protein translation, Ivanova et al. [65] found that *E. coli* enables cellular responses to osmotic stress, plasma membrane regulation and the phospholipid biosynthetic process under THz radiation from a broadband synchrotron source of 0.5–18 THz. After THz radiation, deformed outer membrane, membrane perturbations and leakage of cytosol of bacteria were observed (Figure 3f,g). Studies have also been conducted on bacteria other than *E. coli*. Bannikova et al. [66] explored the non-thermal THz effects on the extremophilic bacterium *Geobacillus icigianus* (*G. icigianus*). They observed changes in various metabolic pathways, such as chemotaxis and the synthesis of peptidoglycan and riboflavin, after both short-term and long-term 2.3 THz exposures from an FEL source. Recently, they found that the biological effects on *G. icigianus* were mainly attributed to disturbances in the expression of genes of the copper, iron and zinc homeostatic systems [57]. One advantage of working with thermophilic strains (such as *G. icigianus*) is their inherent resistance to temperature changes, allowing researchers to better isolate the radiation effects from thermal influence. Otherwise, additional temperature monitoring or control is necessary, following procedures described in refs [62,63,64,65]. Most studies have reported only negligible temperature increases during THz irradiation, usually monitored by infrared thermal imaging. However, infrared cameras have limited temporal and spatial resolution. Under pulsed THz irradiation, localized and non-equilibrium thermal fluctuations may occur on timescales and spatial scales that are difficult to resolve experimentally [64]. More attention should thus be given to the calculation, measurement, and control of such subtle temperature variations.

Although the above studies show that THz waves can alter bacterial cellular activities and the inherent mechanisms at the molecular level are still unclear, there is no clear evidence that THz waves cause genetic mutations inside bacterial cells. For instance, Sergeeva et al. [67] explored the potential mutagenic and genotoxic effects of 2.3 THz radiation on bacteria, concluding that THz waves do not pose harm to bacterial cells. In a more comprehensive study, Shirato et al. [68] employed the Ames test (a widely employed method of evaluating the potential of a stimuli to cause DNA mutations of bacteria) on five different bacterial strains, subjecting them to various stimuli, including a 1.6 THz pulse laser from a parametric generator (3.8 mW/cm^2^), ultraviolet (UV) radiation, and chemical stimulants. Their findings consistently indicated that THz radiation did not exhibit mutagenic properties, nor did it inflict DNA damage. In contrast, obvious DNA damage and low cell viability were observed after UV radiation and chemical stimulants. Therefore, the photon energy of THz waves is confirmed to be insufficient to break molecular bonds and cause genetic mutations. THz waves are more likely to interact with the gene duplication, transcription or translation process to up/downregulate some biomolecular activities.

While most researchers have focused on bacteria, studies on the effects of other species have also been reported. Hadjiloucas et al. [69] conducted preliminary tests on yeast cells, a type of fungi, exposing them to radiation of 200–350 GHz. They observed an increased growth rate, specifically at 341 GHz. In another study, Goryachkovskaya et al. [60] found that the expression levels of 16 proteins in Archaea were altered when exposed to THz radiation. Additionally, diatom algae, a type of phytoplankton with a protective siliceous layer (known as a frustule), showed a unique response that the separation of frustules from a diatom’s cell membrane can be promoted by submillimeter-wave radiation [70].

Although diverse experimental conditions and evaluation parameters have been employed, results from different research groups collectively provide strong support for the existence of non-thermal THz radiation effects on microorganisms. Because THz waves can interact with a wide range of molecular motions, the underlying mechanisms are likely to be complex. Several possible mechanisms have been proposed or identified at the molecular level. For example, phospholipid bilayers exhibit collective vibrational modes in the THz range [71], and irradiation near 3.1 THz has been reported to enhance cell-membrane fluidity, suggesting that resonant excitation of collective lipid motions may alter membrane dynamics and function. Because the dynamics of hydration water surrounding biomolecules overlap with the sub-THz frequency range, Sugiyama et al. showed that THz irradiation may selectively perturb these interfacial water dynamics [15]. Notably, 0.1 THz irradiation was shown to non-thermally accelerate protein hydration and modify the associated hydrogen-bond network. In the study by Tang et al., molecular-dynamics simulations suggest that THz electric fields can induce water polarization and transient water bridges across lipid bilayers [72], providing a possible pathway toward field-assisted electroporation and enhanced membrane permeability.

**Table 2:** Experimental conditions for investigating the THz biological effects in microorganisms.

Microorganism Types		THz Source	THz Type	Radiation Frequency (THz)	Intensity (mW/cm^2^)	Exposure Duration	Temperature (°C)	Biological Effects	Ref.
Bacteria	*E. coli*	FEL	Pulsed, pulse duration 40–100 ps	2.3	1400	15 min	/	Enhanced cell aggregation and cell adhesion; weakened cell division	[62]
	*E. coli*	FEL	Pulsed, pulse duration 2 ms	3.1	33	8 h	37	Increased copy number of plasmids and protein production	[63]
	*E. coli*	FEL	Pulsed, pulse duration 5 ps	4.0 ± 1.0	/	90 s	Room temperature	Significantly affects transcription process	[64]
	*E. coli*	Synchrotron	Pulsed, pulse duration 0.23 ns	0.5–18	0.1	10–90 min	24.57 ± 0.12	Deformed outer membrane, membrane perturbations and leakage of cytosol	[65]
	*Geobacillus icigianus*	FEL	Pulsed, pulse duration 50 ps	2.3	230	15 min	60 ± 1	Various metabolic pathways affected (including cell growth, chemotaxis, etc.)	[66]
	*E. coli*	FEL	Pulsed, pulse duration 50 ps	1.5, 2.0 and 2.3	1400	15 min	35 ± 2	Enhanced protein expression (*katG* gene biosensor)	[58]
	*E. coli*	FEL	Pulsed	2.3	1400	15 min	35 ± 2	Enhanced protein expression (*copA* gene biosensor); no effect on *emrR* gene biosensor	[73]
	*E. coli*	FEL	Pulsed, pulse duration 100 ps	2.3	~140	15–30 min	36 ± 1	Enhanced protein expression (*matA*, *safA* and *chbB* gene biosensor)	[59]
		IMPATT-diode	CW	0.14	~2.0		~26		
	*E. coli*	FEL	Pulsed, pulse duration 100 ps	2.3	140 (cuvette)/180 (microplate)	15–30 min	35–37	Enhanced protein expression (*tdcR* gene biosensor)	[74]
		IMPATT-diode	CW	0.14	2.0	15–30 min	26		
	*E. coli* and *Salmonella typhimurium*	FEL	Pulsed, pulse duration 50 ps	2.3	1400	5–15 min	/	No mutagenicity and genotoxicity; positive effects on cell metabolism	[67]
	*Salmonella typhimurium* and *E. coli*	THz parametric generator	Pulsed	1.6	3.8	20–60 min	37	No mutagenicity and DNA damage	[68]
	*E. coli*	/	/	/	/	15 min	/	No impact on viability and antimicrobial resistance	[75]
	*Bacillus subtilis*	Gunn oscillator	/	0.094	1.3	1–24 h	25	No effect on metabolic activity or population density	[76]
	*E. coli*	THz gas laser	Pulsed, pulse duration 100 ns	4.5	/	50–500 s	/	Cell death at a value of total energy of ~6 J	[77]
Yeast	*Saccharomyces cerevisiae*	Backward wave oscillator	CW	0.19–0.34	~5.78	30–150 min	25	Enhanced growth rate	[69]
Archaea	*Halorubrum saccharovorum*	/	/	2.3	800	5 h	/	Various protein expression levels changed	[60]
phytoplankton	Diatom algae	FEL	Quasi-continuous, pulse duration 30–100 ps	5.6 MHz (submillimeter wave)	20,000	3–10 s	/	Splitting of diatom frustules without destruction of cell content	[70]

### 2.2. Utilization of Biological Effects

**Figure 4:**
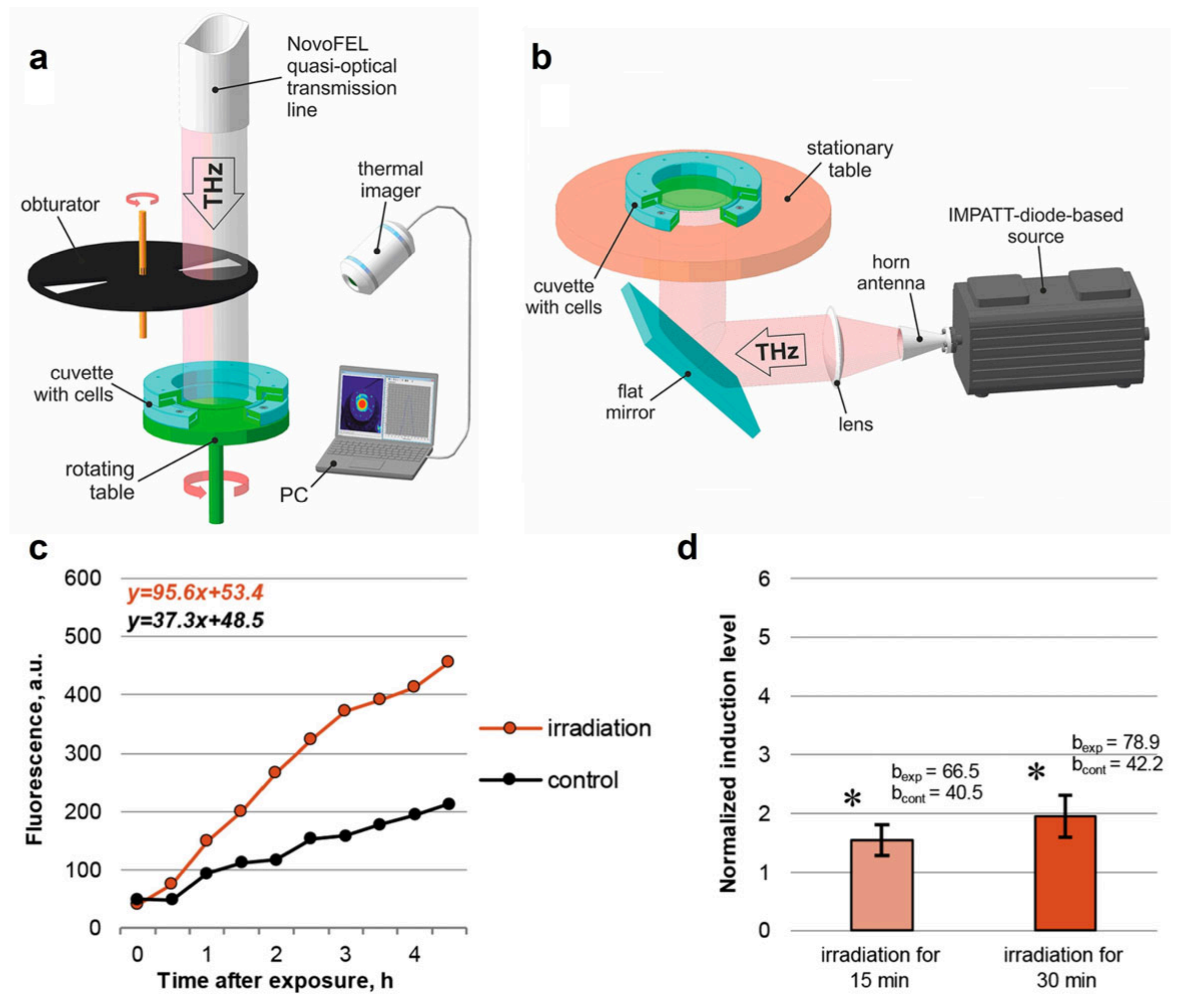
Experimental setup used for *E. coli* radiation based on (**a**) THz FEL source (labeled as NovoFEL, Novosibirsk FEL) and (**b**) IMPATT-diode source. Panels (**a**,**b**) are reproduced with permission from Serdyukov et al., Biomed. Opt. Express 11, 5258–5273 (2020) [59]. Copyright 2020 Optical Society of America. (**c**) Dynamics of fluorescence in response to 30 min THz irradiation in comparison with a control (results of one independent replicate are presented). Equations indicate the linear regression. (**d**) Normalized induction levels (average values from six biological replicates) at 4.5 h after the exposure. The error bars represent standard deviation; b_exp_ and b_cont_ are the average slope coefficients in experiment and control, respectively. * Significant differences (*p* < 0.05) in slope coefficients between experiment and control. Panels (**c**,**d**) are reproduced with permission from Serdyukov et al., Biomed. Opt. Express 12, 705 (2021) [74]. Copyright 2021 Optical Society of America.

In addition to studying the biological effects induced by THz radiation, scientists have utilized bacteria as THz-sensitive biosensors to examine metabolic pathways within bacterial cells. Note that biosensors (short for ‘biological sensors’) have a different definition to THz optical sensors for sensing biomedical analytes. Here, biosensor refers to bacteria with proper transducers that response to targeted substances by producing easily measured physicochemical signals under THz stimulus. In other words, the bacteria function as sensors to respond to other analytes. In contrast, the optical sensors that will be introduced in Section 3 are optical devices that sensitively change their THz response with loaded bacteria or their components (e.g., protein or DNA). Many of these biosensors are based on the bacterium *E. coli* [78,79,80,81], due to its well-known genetic information. For example, through genetic engineering, genes that are sensitive to heavy metals can be linked with genes that express fluorescence in a plasmid. This allows bacteria-based biosensors to detect heavy metals by observing bacterial fluorescence. Peltek et al. have been working on building *E. coli* biosensors to interact with THz waves [58,59,73,74]. Two THz radiation sources have been used in their studies, including a Novosibirsk FEL source and an IMPATT-diode semiconductor source, as shown in Figure 4a,b. Their approach involves using a plasmid containing a promoter and a reporter gene. The promoter is a DNA sequence that initiates DNA transcription, and the downstream reporter gene is a DNA sequence to be transcribed (e.g., the green fluorescent protein, GFP). THz waves can interact with the promoter and increase the expression level of the reporter gene. This setup allows the observation of specific gene expressions in real time and in situ when exposed to THz radiation. For example, promoter *katG* gene encodes for hydroperoxidase I, which protects aerobic and phosphate-starved cells from oxidative damage [82,83]. pKatG-GFP can thus act as a specific indicator for oxidative damage to *E. coli*. Demidova et al. [58] transformed plasmid pKatG-GFP into *E. coli* and found bacteria expressed more GFP with THz radiation. The increased GFP indicated higher expression of hydroperoxidase I, which is the primary catalase in the hydrogen-peroxide-degrading metabolic pathways in bacterial cells [84]. This *E. coli* biosensor can be used to study the metabolism dynamics of *katG*-related genes. Furthermore, the promoters of other sensor genes (including *copA*, *emrR*, *matA*, *safA*, *chbB* and *tdcR*) have been used to investigate different metabolic pathways [59,73,74,84], showing similar increasing gene expressions under THz radiation (Figure 4c,d).

## 3. Detection of Microorganisms Using THz Waves

Although many accurate and target-selective detection methods for microorganisms are readily available, such as quantitative polymerase chain reaction (qPCR), they are generally time-consuming, not in situ, destructive, and often require experimental expertise [85,86]. Spectroscopy is a promising tool for analyzing chemical and biological matter non-invasively and rapidly, which commonly uses frequencies spanning from ultraviolet to infrared. THz waves lie at the far-infrared range that provide unique information of long-range molecular interaction, including hydrogen-bonding networks and intermolecular vibrations of proteins and DNA. Therefore, THz spectroscopy can serve as a novel approach for fast, non-destructive and quantitative detection [16]. With the assistance of field-confining sensors, trace analytes with thickness far smaller than the THz wavelength can be accurately measured. In combination with near-field imaging, spatial resolution down to tens of nanometers can be reached [9]. In this section, we will focus on the detection of microorganisms using THz technologies.

### 3.1. Instrumentations and Sample Preparation

#### 3.1.1. THz Systems

**Figure 5:**
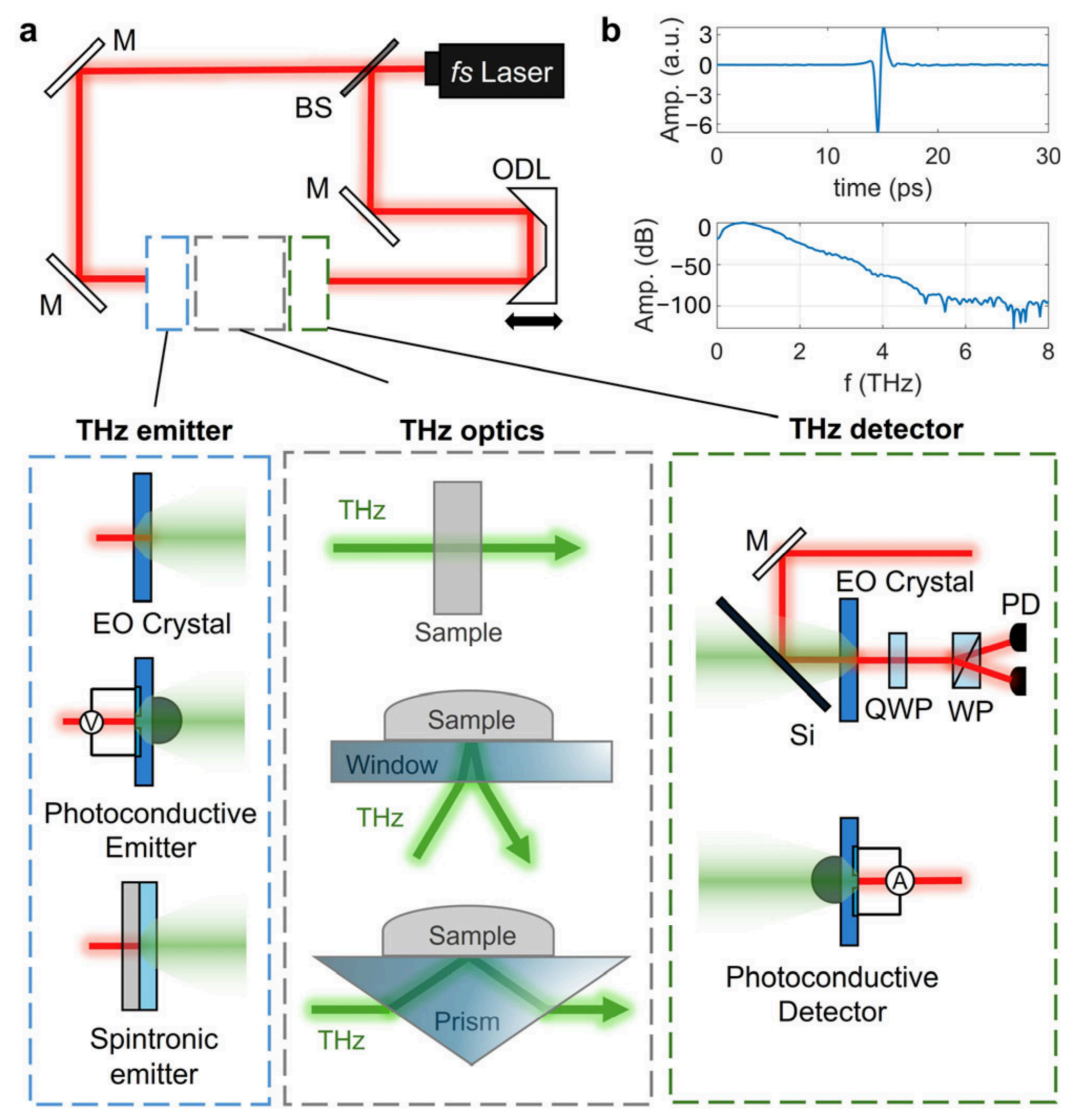
(**a**) Schematic of a typical THz-TDS system and its potential combinations of different THz emitters, detectors and THz optical configurations. (**b**) Example of a THz-TDS signal in time domain (**upper panel**) and frequency domain (**lower panel**). M: mirror, BS: beam splitter, ODL: optical delay line, EO: electro-optic, Si: silicon, QWP: quarter-wave plate, WP: Wollaston prism, PD: photodetector.

THz systems can be classified into pulsed time-domain THz systems and continuous-wave (CW) THz systems. The former are often known as THz time-domain spectroscopy (THz-TDS) systems, which generate and detect THz radiation via photoconductive antennas, non-linear crystals or spintronic devices excited by femtosecond infrared light. Figure 5a shows the schematic of a typical THz-TDS system and its potential combinations with different emitters (e.g., photoconductive antennas, electro-optic crystals and spintronics) and detectors (e.g., photoconductive antennas, electro-optic crystals). The picosecond-scale single-cycle THz electric field is detected in the time domain, as shown in the upper panel of Figure 5b. Fourier transforming this time-domain signal results in a broadband THz spectrum, as shown in the lower panel of Figure 5b. THz-TDS systems are characterized by their picosecond time resolution, ultrabroad bandwidth and coherent detection that provides both magnitude and phase information simultaneously. CW THz systems have various source–detector combinations. For example, THz radiation can be generated by a Schottky diode or backward-wave oscillator and detected by a Golay cell or bolometer. They usually have narrow linewidth and high output power, hence providing better spectral resolution and deeper penetration depth. A comprehensive overview of THz instrumentation is beyond the scope of this review but can be found in other literature [9].

Both pulsed and CW THz systems can be built in two fundamental configurations of transmission or reflection, and both configurations have been used in microorganism detection, as shown in Figure 5a. In transmission, THz beams transmitting through the holder or the device are measured with and without the investigated sample. The two signals are compared and analyzed to extract the intrinsic sample properties. Biological samples are usually hydrated. Their high water concentration strongly absorbs THz light and limits the use of transmission configuration to thick samples. In this case, a reflection configuration can be used. Samples are usually placed on a flat supporting medium, such as a window or a prism, to establish a well-focused interface. Amplitude and phase change of the reflected signal records the sample THz optical information.

#### 3.1.2. Sample Preparation

Microorganisms naturally exist in single-cell or colony form. A single microbial cell floats in liquid medium or adheres to surfaces without contacting other cells, while in the colony form cells are densely packed and filled with extracellular polymeric substances (EPSs) [87]. Microorganism growth often requires a humid or aqueous environment. Unfortunately, water is one of the most absorbing materials in the THz regime, hence samples are usually dehydrated or prepared as thin films for THz measurements [9]. For example, single-cell bacteria are directly deposited on the sensor surface and left to dry. A bacterial colony is scraped from the agar surface and placed into a defined-height chamber for THz measurements. Heating dehydration may be applied to further reduce the water content. The drying process may destroy the original structure of the microorganisms, making it no longer in situ and non-destructive. Freeze-drying is another frequently used method to reduce the absorption influence of water. This approach better maintains the original cellular structures. By employing a reflection configuration, samples can be measured in highly hydrated states without considering the attenuation within the sample. Reflection is especially suitable for samples in a liquid phase that can make intimate contact with a supporting medium. Some microbial samples are naturally in a form appropriate for THz measurements. For example, leaf mildew is a disease of plant leaves caused by microorganisms [88]. The thin leaf (hundreds of microns) allows the penetration of THz waves, enabling distinguishing infected and non-infected areas based on their water content difference.

### 3.2. THz Spectroscopy and Imaging of Microorganisms

#### 3.2.1. THz Spectroscopy of Microorganisms

As the extension of infrared spectroscopy, the photon energy of the THz waves matches the energy levels of low-frequency biomolecular vibrations originating from intra- and inter-molecular weak interactions. It is expected that these molecular motions may specifically respond to some of the THz frequencies to produce characteristic absorption features. However, they cannot be observed in targets with complex molecular compositions such as microorganisms, which has been explicitly discussed in the perspective by Markelz and Mittleman [89]. In detail, the intermolecular oscillations of small molecules can only produce observable features in the crystalline form from their phonon resonance, which are the result of the long-range ordering structure. Biological macromolecules have a high density of states which result in featureless continua. Aqueous samples are characterized by the continuous and strong absorption of a highly damped hydrogen-bonding network. Studies on amorphous small molecules [90,91], molecular solutions [92,93,94] and biological macromolecules [95,96] have validated the above physical predictions. For samples like microorganisms which contain a huge number of different molecules and are often in an aqueous state, there remains no possibility of observing any characteristic fingerprint absorptions.

Indeed, Johnson et al. [97] measured the mid-IR and far-IR spectra of five *Bacillus* strains. They found characteristic frequencies in the mid-IR range but no signature in the THz range (far-IR). Tang et al. [98] investigated the spectral response from 0.2 to 2.2 THz of *Bacillus* spores. Similarly, no THz signatures were observed for either bacterial cells or their main chemical components. The authors explained that the monotonically increasing absorption with THz frequency was mainly contributed by Mie scattering and remnant water. Other bacteria have also been studied [99,100]. Although the slope of the absorption–frequency curve differs in different bacterial samples, there were no characteristic absorption peaks that can be used as identification labels. In studies of halophilic archaea, there is no signature in the absorption–frequency curve of the main component bacteriorhodopsin [101,102]. Due to the lack of sample-specific features, direct microorganism identification by THz spectroscopy is challenging.

It should also be mentioned that some studies claim to have observed characteristic absorption of microorganisms in the THz band, including *E. coli* cells [103,104,105] and *Bacillus* spores [104,105,106,107,108]. Note that these reports are only from two groups. These studies suggested that genetic materials [103,105,109] or the major cellular component (e.g., dipicolinic acid) [110] could be the source of the signatures. However, these explanations contradict condensed matter theory. These works used frequency-domain THz systems, which exhibit dense oscillations in the spectrum originated from the standing-wave effect that can be mistakenly regarded as characteristic absorptions. The observed peaks could also be induced by noise, water-vapor absorptions or cavity-like oscillations, which should be carefully removed or calibrated to extract the intrinsic sample information, as highlighted in ref [89].

THz spectroscopy has been further explored for water pollution evaluation. Microalgae are unicellular photosynthetic eukaryotes; they usually live in fresh water and oceans, producing approximately half of the oxygen on Earth. Except for providing valuable metabolites like carotenoids, microalgae have emerging potential in bioremediation. For example, investigating the effects of heavy metal pollution (such as lead ion (Pb^2+^) pollution) on microalgae provides important information for water quality management and algal bioremediation [111]. However, metabolite quantification of microalgae requires using several time-consuming techniques at the same time, such as chromatography and spectrophotometry. A simple, rapid and low-cost detection method is demanded. Because the main algal metabolites (including β-carotene, astaxanthin and starch) produced absorption signatures in the far to mid-IR regime, the group led by Prof. Peng Yan has conducted a series of studies using an FTIR spectrometer (0.9–20 THz) to quantify metabolites in algae in real time [112,113,114]. Generally, photosynthesis is inhibited under Pb^2+^ stress and cells prefer to synthesize less-complex biomolecules (such as carbohydrate and carotenoid), rather than proteins. The measured THz characteristic frequency peaks correlate to the metabolite concentrations. Their results showed that the storage of carbohydrate and carotenoid was facilitated. Based on the THz spectral data, the concentration of heavy metal ions can be well predicted by the established model with high accuracy, high efficiency and small amounts of sample. A similar technique was also applied in the heavy metal detection of soil [115]. These findings demonstrate the potential of using THz spectroscopy as a fast and non-destructive tool to evaluate the level of environmental pollution.

#### 3.2.2. THz Imaging of Microorganisms

**Figure 6:**
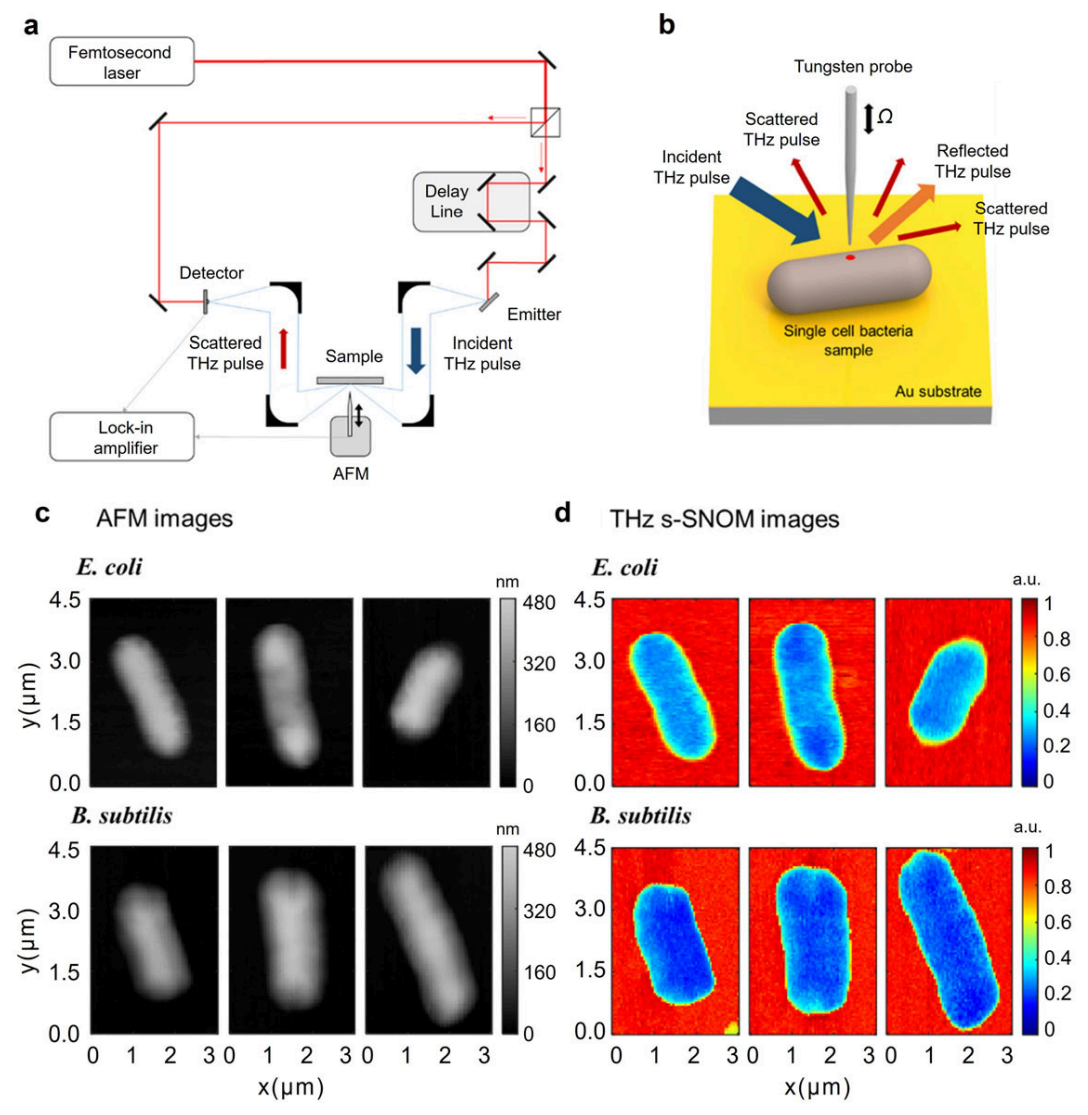
(**a**) Schematic of the THz s-SNOM experimental setup, which comprises a THz-TDS system integrated with an AFM. (**b**) Illustration of the THz near-field interaction involving the oscillating tungsten probe tip and a bacterial cell on a Au substrate. THz. (**c**) AFM topography maps of *E. coli* and *B. subtilis* on an Au substrate. (**d**) THz s-SNOM images acquired simultaneously with the AFM images. Pixel values represent the peak intensity of the scattered THz pulse measured in the time domain. Reproduced with permission from Lee et al., J. Biomed. Opt. 30, 1–13 (2025) [117]. Copyright 2025 the authors, licensed under a Creative Commons Attribution (CC BY) license.

Because of the subwavelength structures, THz imaging of microorganisms has been mainly realized by THz near-field techniques, such as with a THz scattering-type scanning near-field optical microscope (THz s-SNOM) [116,117,118,119,120]. Figure 6a,b illustrate the configuration and operation principle of THz s-SNOM [117]. This technique uses a nanometer-scale tip in an atomic force microscope (AFM) to scatter the incident THz field. The tip oscillates at a frequency of *Ω* such that the scattered THz field is also modulated at this frequency. By demodulating the detected THz signal at the harmonics of *Ω*, near-field THz signals can be retrieved from the intense far-field reflection background. By scanning samples over a certain area, THz images and AFM morphological images can be simultaneously acquired with a resolution of tens of nanometers, four orders of magnitude smaller than conventional far-field THz images. The intrinsic local dielectric properties of samples can be extracted from the THz signals by using an appropriate electrodynamic model, such as the finite dipole model (FDM) or extended dipole model. In these models, the AFM tip is approximated as a polarizable spheroid or dipole, and the tip–sample near-field interaction is described self-consistently through the sample dielectric response. By combining the measured near-field amplitude and phase with a reference and numerically inverting the model, the local complex dielectric permittivity can be quantitatively extracted, extending THz s-SNOM from qualitative contrast imaging to quantitative mapping of subcellular structures.

Figure 6c,d show the corresponding AFM and THz s-SNOM images of *E. coli* and *B. subtilis* [117]. Although their morphological features are similar in the AFM results, their THz responses are different owing to their different THz dielectric properties. This example shows the capability of rapidly differentiating different bacterium species from THz near-field images. Based on these characteristics, THz s-SNOM has been successfully applied to distinguish *E. coli* from *S. aureus* [116], enabled by the lower dielectric constant of *E. coli* which leads to a weaker scattered light. The observation can be well explained by the proposed finite dipole model. The group further investigated antibiotic susceptibility test of *S. aureus*. Bacterial strains that are resistant to and susceptible to cell-wall-inhibiting antibiotics produced distinct near-field signals. The classifying results obtained from THz-SNOM match well with traditional antibiotic susceptibility tests, while it significantly reduces the time cost by its culture-free and label-free mechanism, presenting promising potential in drug-resistant bacteria screening. However, we should also point out that current THz s-SNOM experiments have only been applied to dehydrated samples. The strong water absorption prevents direct signal acquisition from aqueous environments. In addition, the reduced AFM tapping stability, difficulties in catching weakly adhered microorganisms in liquids, and potential pollution introduced to the tips are all challenges yet to be overcome.

**Figure 7:**
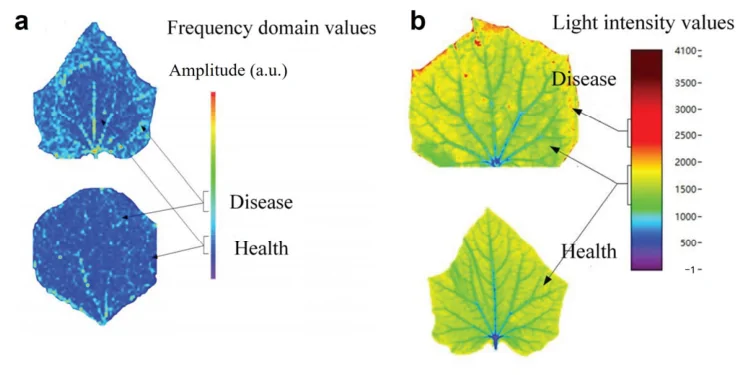
(**a**) THz characteristic images and (**b**) NIR characteristic images at 1395 nm of cucumber powdery mildew. Reproduced with permission from Zhang et al., Front. Plant Sci. 13, 1035731 (2022) [88]. Copyright 2022 the authors, licensed under a Creative Commons Attribution (CC BY) license.

Alternatively, far-field THz imaging can be performed for large samples, the status of which is related to microorganism activities. For example, crop diseases, mainly caused by fungal pathogens, significantly affect the yield and quality of agricultural products, which are highly related to food safety and human health. THz technology has been applied in the field of plant disease diagnosis. Compared with time-consuming and expensive biochemical methods such as PCR and DNA microarray techniques, THz spectroscopy is advantageous in providing a wealth of information with low cost and the capability of real-time in situ batch sampling. A typical example is the identification of fungal infections in chestnuts with 0.1 THz continuous-wave radiation [121]. The husk of a chestnut is a thin and dry shell, and therefore transparent in the THz region, allowing non-destructive measurements without opening the outer shell. Inside the chestnut, the THz signals attenuate with the absorbance of the fruits based on Beer–Lambert’s law. The total attenuation was found to be proportional to the water content, and infected chestnuts have reduced water content due to carbohydrate hydrolysis by fungi. Similar results were also found in hazelnuts [122]. Other plant diseases, including late blight and fusarium infection of potatoes [123], cucumber powdery mildew [88], tomato leaf mildew [124] and infested wood [125], were also investigated. In the study of cucumber powdery mildew, THz waves were able to assess the degree and depth of the diseased plant tissues and differentiate the decayed parts from the healthy parts, as shown in Figure 7. More recently, THz imaging has been further combined with hyperspectral technology and machine vision [88,124,126] to enable a more comprehensive evaluation of plant health with better recognition accuracy.

### 3.3. THz Sensing of Microorganisms

#### 3.3.1. THz Sensors

**Figure 8:**
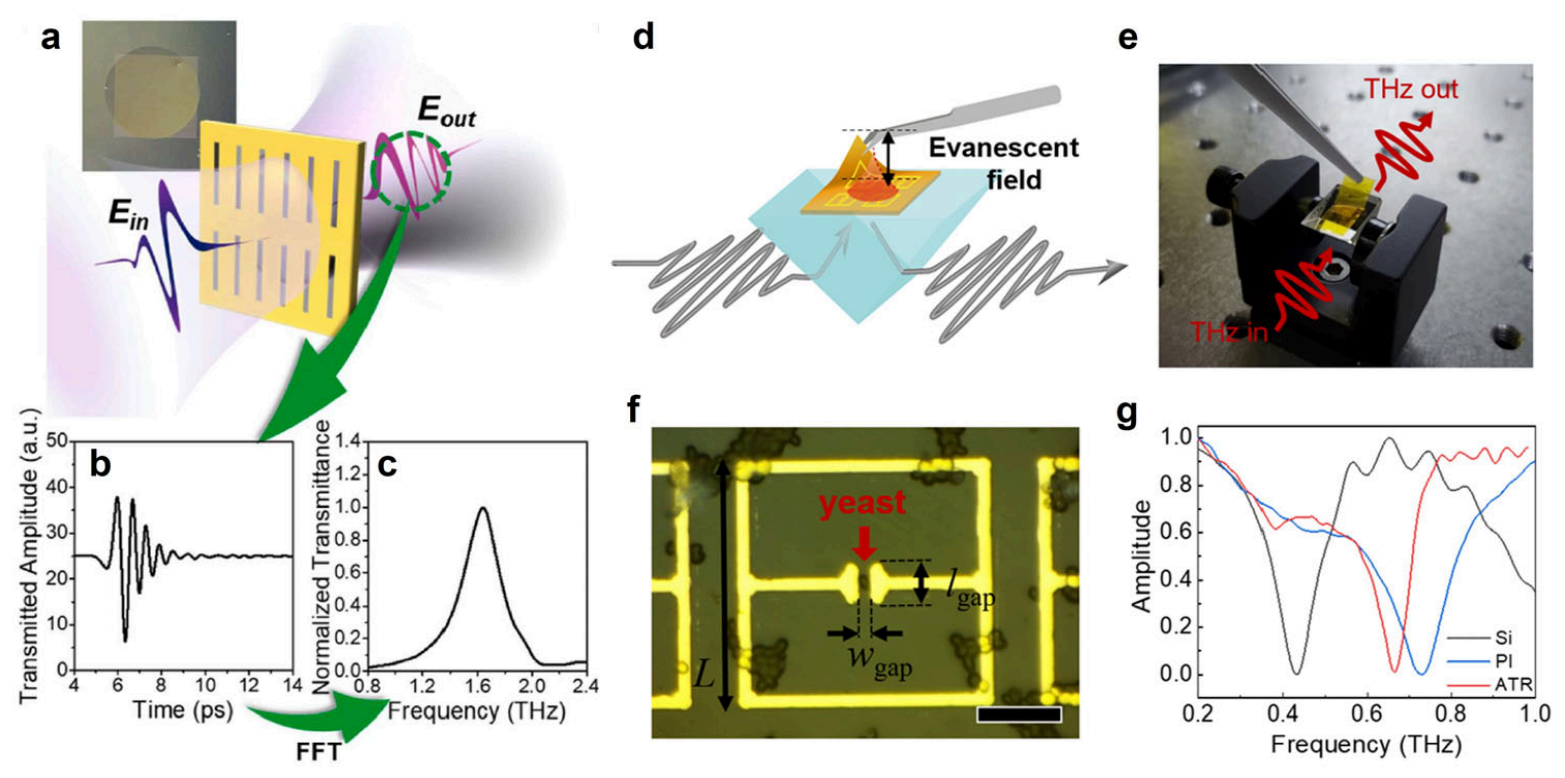
Examples of THz MM sensors for enhancing the detection sensitivity of microorganisms. (**a**) Schematic of the nanoslot-array MM sensor. (**b**) Time-domain waveform and (**c**) frequency-domain spectrum of the THz signal transmitted through the sensor. Panels (**a**–**c**) are reproduced with permission from Lee et al., Biosens. Bioelectron. 202, 113981 (2022) [127]. Copyright 2022 the authors, licensed under a Creative Commons Attribution-NonCommercial-NoDerivatives (CC BY-NC-ND) license. (**d**) Schematic of a thin-film MM sensor coupled with a prism. (**e**) Photograph of the MM–prism structure. (**f**) Microscope image of the split-ring resonator patterns of the sensor with yeast deposited on it. (**g**) Normalized THz transmission spectra through the metamaterials fabricated on a Si substrate (black), flexible PI (polyimide) film (blue), and through the hybrid MM–prism device (red). Panels (**d**–**g**) are reproduced with permission from Kim et al., Opt. Express. 32, 48915–48924 (2024) [130]. Copyright 2024 the authors, licensed under a Creative Commons Attribution (CC BY) license.

One of the biggest challenges of detecting microorganisms using THz waves is the low light–sample interaction efficiency due to the scale mismatch between the THz wavelengths and the sample size. The micrometer-scale size of microorganisms is over an order smaller than the submillimeter wavelengths of THz waves, resulting in weak changes to the THz signal that can be easily affected by noise and measurement errors. Therefore, sensors capable of enhancing sample-interaction efficiency are highly demanded. This is typically achieved by field-confining techniques. Metamaterials (MMs) are the most widely used type [127,128,129,130,131], which contain artificial subwavelength periodic structures resonating at specific frequencies. When MMs are fabricated in a planar form, they are usually termed as metasurfaces. Figure 8 shows two examples of THz MM sensors, including a nanoslot sensor operated in transmission (Figure 8a–c) and a split-ring resonator (SRR) sensor operated in reflection by coupling to a prism (Figure 8d–g). The fundamental mechanism is similar for different pattern designs, which aims at confining the THz electric field to a height of only a few micrometers. Such a design not only perfectly matches the scale of microorganisms but also significantly enhances the interaction efficiency and detection sensitivity. The sensor resonance is narrowband that leads to a sharp peak or dip in the spectrum. Analyte loading sensitively shifts the resonant positions, enabling quantification by the amount of frequency shift or amplitude change. Apart from MMs, other strategies have also been reported, such as utilizing the evanescent mode of a suspended core THz fiber [132] or by using a metallic mesh sensor [133], antenna [134], thin-film enhancement [135] or THz surface plasmon polaritons (SPPs) [136,137].

We summarize MM-based sensors for microorganism detection in Table 3 due to their widespread application. A common architecture is fabricating the metallic MM structure on THz-transparent substrates of silicon, quartz or polymer. Some additional techniques may be applied to further enhance the sensitivity, such as in combination with rolling circle amplification (RCA) [138], nanoparticles [139], graphene and nanoantenna structures. For sample preparation, most experiments simply adopt the droplet deposition–dehydration process, whilst some studies functionalized the sensor with specific antibody to selectively detect targeted microorganisms, especially viruses. The resonance with specific THz frequencies is reflected as resonant peaks or dips in the spectrum, depending on the sensor types and optical configurations. The red shift of the resonant frequency and the decay of the resonant strength are the typical responses of MMs to the loaded microorganisms, due to the increased dielectric constant and the damping effect caused by the increased absorption. As such, quality factor (Q-factor) and sensitivity are the two most important parameters to evaluate sensor performance. The former is defined as the ratio of the resonant frequency over the resonance width (typically full width at half maximum, FWHM). A higher Q-factor leads to a sharper peak or dip, enabling a more accurate readout of the resonant frequency. The sensitivity is usually defined as the frequency shift per unit refractive index change (RIU), or sometimes per unit volume/concentration. Note that these parameters should be evaluated together to estimate the general performance, and one should notice that there are usually big differences between the simulation results and the practical values. For example, a Q-factor up to a few hundred or even higher can be achieved in an ideal simulated structure, while practically a Q-factor beyond 100 is challenging due to the limited fabrication accuracy, ohmic loss in metals and absorption loss in substrate. Q-factors are further damped when sensing hydrated samples due to the strong water absorption. Some studies have reported sensitivity over 1 THz/RIU at a very high frequency according to the simulation, which could be challenging to achieve since most THz systems cannot provide sufficient dynamic range at these frequencies. As listed in Table 3, the performance of a sensor can also be evaluated by its limit of detection (LOD), which is determined not only by the intrinsic sensor performance but also influenced by factors such as sample preparation and the THz measurement procedure. However, the unit used to report the LOD varies depending on the target analyte. For bacteria, colony-forming units (CFU)/mL is the most widely used unit, although cell density (e.g., cells/μm^2^) may also be reported. For biomolecular targets such as RNA, DNA, and proteins, units such as pg/mL or molar concentration (M) are commonly used. Currently, standardized reporting practices for LOD in THz sensing remain lacking. Researchers are therefore encouraged to report LOD using commonly accepted units and consistent calculation criteria for a given target, thereby facilitating quantitative comparisons among different studies.

#### 3.3.2. Sensing Applications

Sensitive detection and identification of microorganisms in the THz regime can be realized with the assistance of THz sensors. Yu et al. [140] functionalized Fe_3_O_4_@Au nanocomposites with a *Staphylococcus aureus* (*S. aureus*)-specific aptamer for bacterial detection. Bacteria–nanomaterial complexes could rapidly separate and enrich the target bacteria in a mixed bacterial solution, and the conjunction of nanomaterial significantly enhanced the resonance frequency shift when the samples are loaded on the THz MM sensor. Bacterial concentration monitoring and an antibiotic susceptibility test can be achieved by microfluidic MMs based on the Fano resonance effect [141]. The detection limit for *E. coli* is as low as 5×10^3^ cells/mL and bacterial concentration under antibiotic treatment could be dynamically obtained. Bacteria-dependent sensitivity of MM sensors has also been reported by Zhong et al. for the identification of *Staphylococcus epidermidis* (*S. epidermidis*) and *S. aureus* [142]. Ma et al. [143] proposed metal–graphene hybrid metasurfaces coupled with polyethylenimine-modified CuS nanoparticles for the detection and in situ inactivation of pathogenic bacteria, achieving a limit of detection down to 11 CFU/mL.

**Figure 9:**
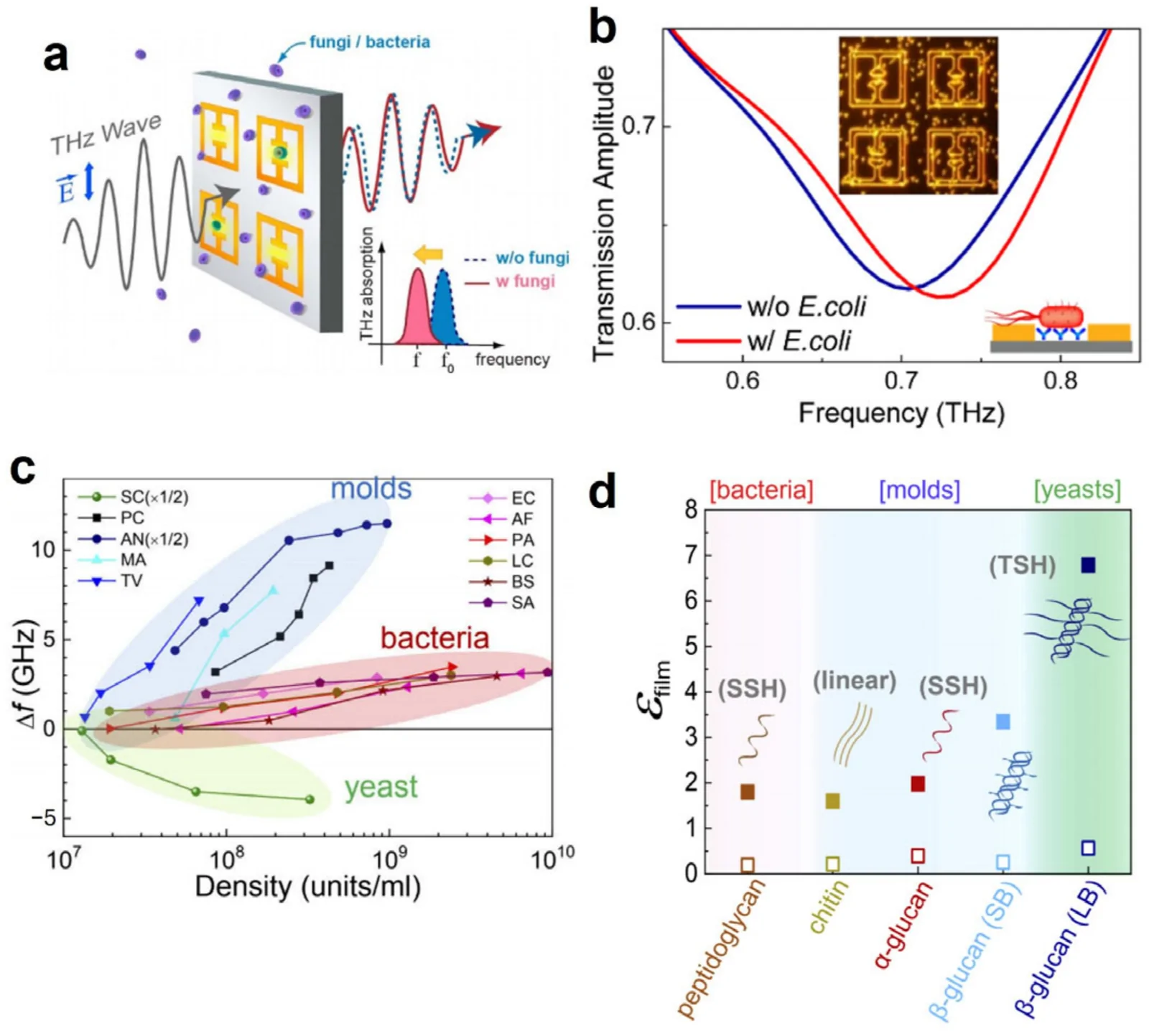
(**a**) Schematic presentation of THz metamaterial sensing of microorganisms. (**b**) THz transmission before (blue line) and after (red line) the deposition of *E. coli* on the functionalized metamaterials in aqueous environments. (Inset) Corresponding dark-field microscopic image obtained after the deposition of *E. coli*. Panels (**a**,**b**) are reproduced from Park et al., Sci. Rep. 4, 4988 (2014) [131], Copyright 2014, licensed under a Creative Commons Attribution-NonCommercial-ShareAlike 3.0 Unported (CC BY-NC-SA 3.0) License. (**c**) Plot of frequency shift as a function of number density for different species. (**d**) Real (filled boxes) and imaginary (open boxes) parts of dielectric constants for peptidoglycan and polysaccharides films, measured at 1 THz. SC: *S. cerevisiae*, PC: *P. aeruginosa*, AN: *A. niger*, MA: *M. ambiguus*, TV: *T. viride*, EC: *E. coli*, AF: *A. faecalis*, PA: *P. aeruginosa*, LC: *L. casei*, BS: *B. subtilis*, SA: *S. aureus*, SSH: single-stranded helix, TSH: triple-stranded helix. Panels (**c**,**d**) are reproduced with permission from Yoon et al., Biomed. Opt. Express 11, 406 (2019) [144]. Copyright 2019 Optical Society of America.

**Figure 10:**
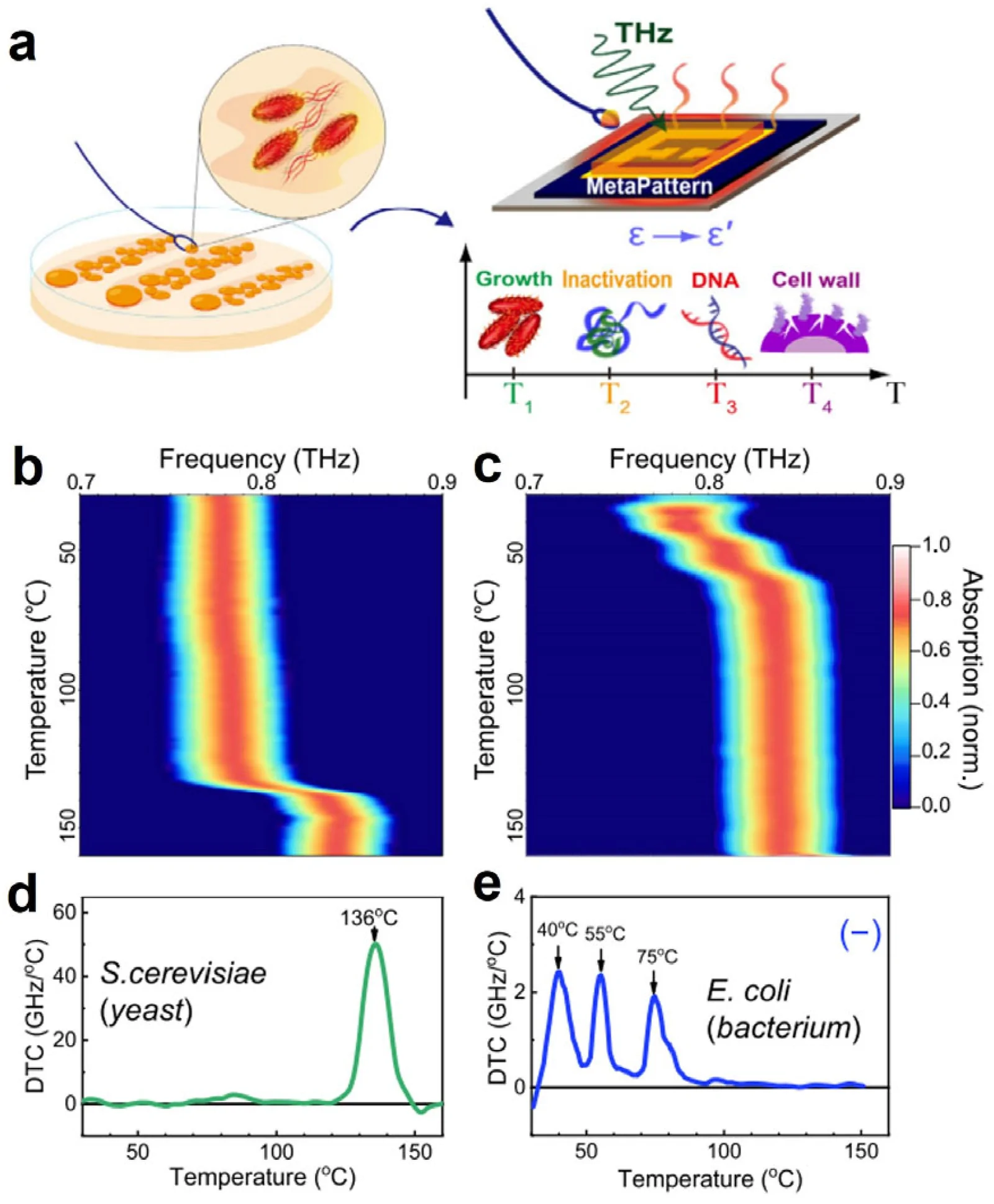
(**a**) Schematic of the THz experiments. The microbial films grown on a culture medium are transferred to THz metamaterials on a ceramic heater. The ceramic heater is punctured at the center with a diameter of 2 mm to enable the transmission experiments. The microbes exhibit phase change with increasing temperature in conditions of growth, inactivation, DNA denaturation, and cell wall destruction. 2D plot of THz absorption through metamaterials coated with a (**b**) yeast layer (*S. cerevisiae*) and (**c**) *E. coli* layer as functions of THz frequency (x-axis) and temperature (y-axis). Differential thermal curves for (**d**) *S. cerevisiae* and (**e**) *E. coli* obtained from panels (**b**) and (**c**), respectively. Minus sign (−) in (**e**) indicates that *E. coli* is Gram-negative bacteria. Reproduced with permission from Jun et al., Nat. Commun. 13, 3470 (2022) [145]. Copyright 2022 the authors, licensed under a Creative Commons Attribution (CC BY) license.

The group from Ajou University proposes various approaches for the detection of living microorganisms based on THz waves. They fabricated SPR MMs to detect viable microorganisms in aqueous environments by integrating a thin-film liquid cell, including molds, yeast and bacterial cells, with very low surface density (~0.05 cell/μm^2^). Clear resonant frequency shifts were observed due to the change of the effective dielectric constant introduced by the microorganisms (Figure 9a,b) [131], and the detection sensitivity can be further enhanced by employing the attenuated total reflection (ATR) geometry [130]. The chemical composition of the cell wall varies among different microorganisms. Therefore, they subsequently examined the main component in cell walls and found that different peptidoglycans and polysaccharides result in different THz dielectric constants, leading to different MM responses (Figure 9c,d) [144]. Interestingly, in subsequent work they further performed a thermal curve analysis with microorganism-coated MMs and found unique THz fingerprints as a function of temperature [145,146], rather than as a function of frequency. In detail, they heated up the MM sensors loaded with different bacteria and found the resonant frequency shows a sudden change at specific temperatures (Figure 10a–c). These changes become more prominent in the first-derivative plot (Figure 10d,e). Since the shifting temperatures are bacteria-specific, they could be leveraged as unique features to differentiate bacteria of different types. The characteristic temperatures were consistent with reported values measured by other approaches, which were classified into four temperature phases of growth phase, thermal inactivation phase, DNA denaturation phase and cell wall destruction phase. Traditional THz MM sensing only reflects the dielectric difference at the resonant frequency. This work further took the thermal dimension into consideration. Pathogenic bacteria were easily distinguished from the bacterial mixture from the thermal curves. The work shows promising potential in bacterial identification in clinical and environmental settings.

**Figure 11:**
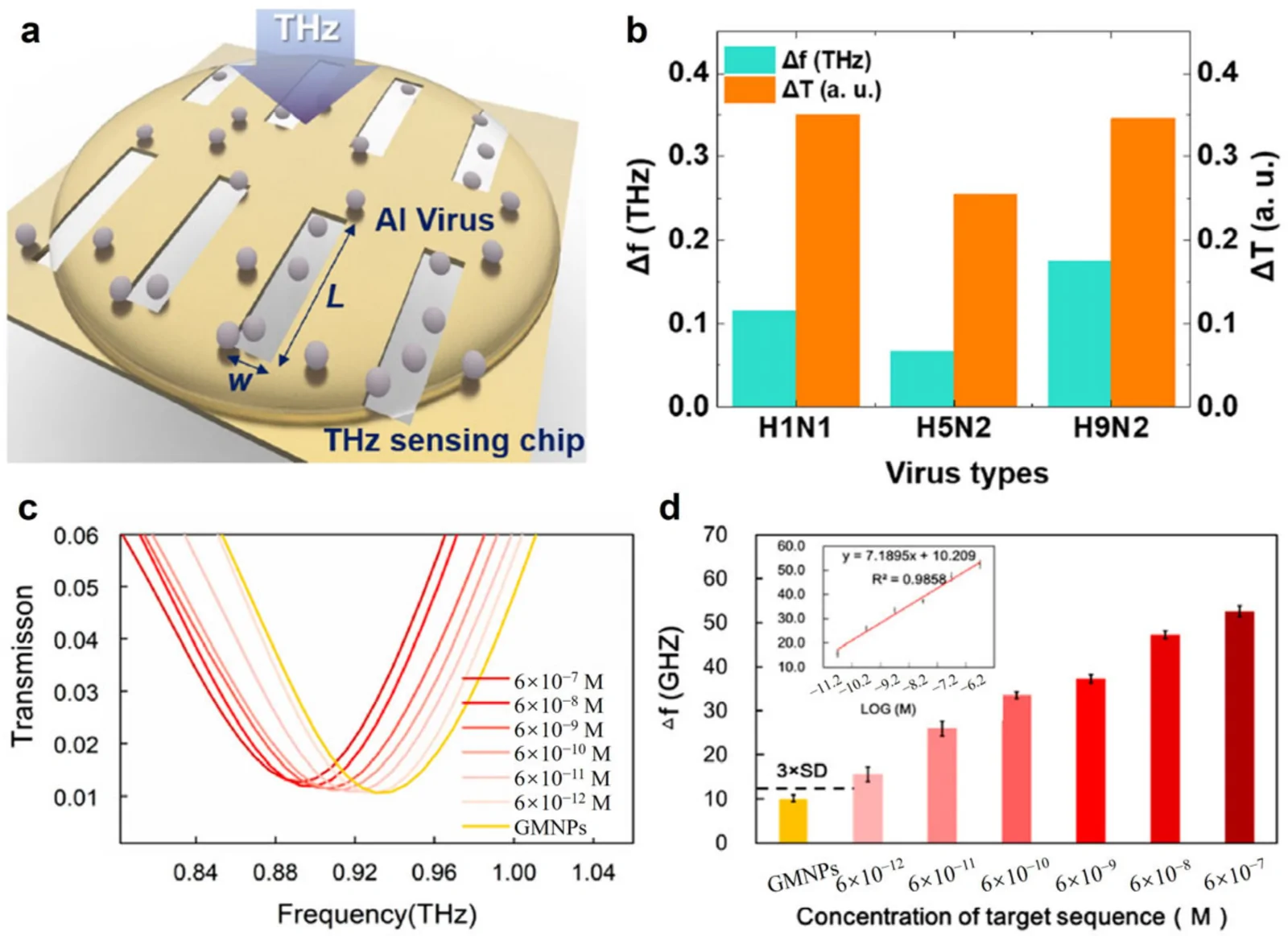
(**a**) Schematic of THz detection of virus samples in liquid state using a nanoslot-antenna-array-based sensing chip. (**b**) Frequency shift and amplitude change of the sensor chip to H1N1, N5N2 and H9N2 samples, respectively. Panels a, b are reproduced with permission from Lee et al., Sci. Rep. 7, 8146 (2017) [148]. Copyright 2017 the authors, licensed under a Creative Commons Attribution (CC BY) license. (**c**) Sensor transmission spectra and (**d**) frequency shifts (Δf) for blank gold magnetic nanoparticles (GMNPs) and different concentrations of the HBV target sequence. Error bars indicate the standard deviation (SD) (n = 3). The inset graph shows the linear fit of the frequencies (Δf) versus the logarithm of the HBV target sequence concentration. Panels c, d are reproduced with permission from Li et al., Front. Bioeng. Biotechnol. 10, 930800 (2022) [129]. Copyright 2022 the authors, licensed under a Creative Commons Attribution (CC BY) license.

Because of the highly similar sensing architecture, we also review THz virus sensing in this section. Virus detection has attracted more attention after the coronavirus disease 2019 (COVID-19) pandemic. Numerous detection methods (e.g., real-time PCR) have been utilized for severe acute respiratory syndrome coronavirus 2 (SARS-CoV-2) detection. However, faster and more accurate methods are continuously demanded, which can minimize the cost of treatment and isolation. THz sensors have also been investigated as a tool for virus identification and characterization, especially for SARS-CoV-2. Sensors are essential [128,147,148,149,150,151] due to the significant scale mismatch between the virus/protein and the THz wavelength. For example, Sengupta et al. [147] fabricated an MM-based chip for coronavirus screening via exhaled breath analysis. The authors claimed that shifts of about 1.5–9 GHz were observed for coronavirus-positive patients, greater than shifts of about 0–1.5 GHz for healthy individuals. However, this method lacks robustness since all kinds of biological particles, including viruses, cytokines, cell debris, etc., can cause red-shifts of the resonance frequency of MMs. Similar principles can be applied for other viruses. For example, Lee et al. [148] used a nanoantenna sensing chip for H1N1, N5N2 and H9N2 viruses, respectively. The nanogaps significantly enhance the field strength to amplify the signal changes induced by the samples. The results show distinct frequency shifts and amplitude reduction for the three viruses, as shown in Figure 11a,b.

Instead of sensing the virus directly, targeting the structural proteins of a virus provides better specificity and robustness and is more widely adopted. SARS-CoV-2 is formed by four major structural proteins. Among them, spike protein has the largest size and is located on the outer surface of the virion. Spike protein is the first area to contact the host cell and responsible for viral entry (binding and fusing) into the host cell, hence it has been considered as an important diagnostic target. THz sensors have also been developed by targeting the spike protein for rapid and precise screening of SARS-CoV-2 [152]. The specific detection of spike protein was achieved by an antibody [153], chemical treatment [154] and direct immersion in protein solution [155]. Derived peptides from the spike protein of different types of SARS viruses can be discriminated even within the same genus [127], showing great specificity. In addition, a low detection limit is demanded for efficient and massive COVID-19 screening. For this purpose, functionalized gold nanoparticles have been used to enhance the antibody binding strength and subsequent detection sensitivity [153]. Magnetic nanoparticles were employed to selectively bind with the spike protein and migrate together towards THz MMs under an external magnetic field [156]. Nanoparticles have also been applied for enhancing specificity and sensitivity of other viruses. In the work by Li et al., hybrid gold magnetic nanoparticle-mediated rolling circle amplification (GMNP-RCA) with gold nanoparticles (AuNPs) was used to form GMNPs-RCA-AuNPs sandwich complexes [129]. By depositing the complexes onto a THz MM sensor, they realized specific and sensitive detection of hepatitis B virus (HBV) DNA, with a limit of detection as low as 127 IU/mL, as shown in Figure 11c,d. Despite the promising accuracy demonstrated in different works, THz virus detections have only been conducted in laboratory settings. The robustness and anti-interference performance in practical disease control applications still need to be validated through more comprehensive large-scale experiments.

**Table 3:** Parameters of MM sensors for microbial detection.

Type	Analytes	Pattern Morphology	Deposition Methods	Performance	Resonant Frequencies	Note	Ref.
Bacteria	*E. coli*	/	Capturing by phages	Limit of detection (LOD) 10^4^ CFU/mL	/	Based on suspended core THz fiber	[132]
		/	Droplet deposition	LOD 10^6^ CFU/mL	/	Based on metallic mesh sensor	[133]
		Double-SRR	Microfluidic device	LOD 5 × 10^3^ CFU/mL, Q-factor 42	0.65 THz	Fano resonance effect	[141]
	*E. coli* *S. aureus*	Metal wire and a pair of SRRs	Droplet deposition	LOD~10^4^ CFU/mL; 378 GHz/RIU; Q-factor 21	1.53 THz	/	[157]
	*S. aureus*	SRR	Specific aptamer binding	LOD 4.78 × 10^2^ CFU/mL	0.8 THz	Aptamer-functionalized Fe3O4@Au nanocomposites	[140]
	*S. aureus* *S. epidermidis*	SRRs and bars	Droplet deposition	556 GHz/cell µm^−2^ and 237 GHz/cell µm^−2^	0.99, 1.1 1.16 THz		[142]
	*Cyanobacteria*	SRR	Droplet deposition	/	0.87 THz	Obtained a differential thermal curve	[146]
	*Mycobacterium*	/	/	Relative sensitivity of 90.6%	/	Based on photonic crystal fiber; only simulation	[158]
		Bow-tie structure	Droplet deposition	1.5 THz/RIU; Q-factor 413	1.9, 2.7 THz	/	[159]
	Five bacterial strains	/	Droplet deposition	/	/	Based on antenna	[134]
	Four bacterial strains	SRR	Droplet deposition	LOD 0.08 pg/mL	0.86 THz	Based on gold nanoparticles and RCA	[139]
	Four bacterial strains	Hollow-core	Deposited on inner surface	/	0.35, 0.5 THz	Based on photonic Bragg fiber	[160]
	Bacterial DNA	/	/	Genomic DNA LOD 0.05 ng/μL	/	Based on rolling circle amplification (RCA)	[138]
		Asymmetry split-ring metasurface	Pyrene group binding	100 nM DNA	0.5 THz	Incorporated with microfluidic device	[161]
Molds, yeasts and bacteria	Molds, yeasts and bacteria	SRR	Specific antibody binding	LOD 10^7^ units/mL	0.84 THz	/	[131]
	14 species of molds, yeasts and bacteria	SRR	Microfluidic channel	/	0.8 THz	/	[144]
	10 species of yeasts and bacteria	SRR	Droplet deposition	80 GHz/RIU	0.77 THz	Obtained a differential thermal curve	[145]
	Yeast	SRR	Droplet deposition	LOD 7 × 10^−3^ cell/µm^2^; Q-factor 6	0.68 THz	ATR geometries	[130]
Viruses	Avian influenza viruses	Jerusalem cross	/	/	1.4, 3.2 THz	Only simulation; based on spoof surface plasmon polaritons	[136]
		Grating split ring resonator	/	300 GHz/RIU; Q-factor 690	1.93 THz	Based on THz surface plasmon polaritons	[137]
		Nanoantenna	Droplet deposition	/	0.62, 0.93, 1.31 THz	/	[148]
		H-shaped	/	540 GHz/RIU	1.72 THz	Only simulation; pattern material is graphene and substrate material is semiconductor	[149]
		Chiral split ring	Specific antibody binding	~4 dB/RIU	1.15, 1.46 THz	Pattern material is graphene	[150]
		Asymmetric split-ring resonators	Droplet deposition	30 GHz/RIU; Q-factor 6	0.4, 0.6 THz	/	[151]
		Nanofake	Droplet deposition	9.2 GHz/RIU	60 THz	Only simulation; pattern material is black phosphorus	[162]
Viruses	Flu viruses, SARS-CoV-2 virus	Star-shaped holes	Droplet deposition	2200 GHz/RIU; Q-factor 19	1.97, 3.37 THz	/	[128]
	SARS-CoV-2 virus	Cross-arrowhead	Breath exhaled	/	0.81 THz	/	[147]
		SRR	/	490 GHz/RIU	2.3 THz	Only simulation; pattern material is graphene	[152]
	SARS-CoV-2 virus spike protein	Toroidal metasurface	Specific antibody binding	LOD ~4.2 fM; Q-factor 14	0.4, 0.6 THz	AuNPs functionalized	[153]
		Three-split ring	Droplet deposition	LOD 5 ng; 73.2 GHz/RIU	0.68, 1.63 THz	/	[154]
		SRR	Immersion	/	0.85, 1.06 THz	/	[155]
	SARS-CoV-2 virus spike protein	Elliptical grooves	Specific antibody binding	LOD 0.002 ng/mL	0.53 THz	Utilized magnetic nanoparticles	[156]
	SARS-CoV-2 spike-protein-derived peptides	Nanoslot arrays	Droplet deposition	LOD 0.1 mg/mL (i.e., 41.7 μM).	1.16/1.64/2.07 THz	/	[127]
	Bacteriophage	SRR	Droplet deposition	70 GHz/RIU	0.8/1.2 THz	200 nm gap	[163]
		Nanogap-loop array	Droplet deposition	/	0.77 THz	Virus-sized nanogap	[164]
		Hybrid slot antenna	Spin-coated	32.7 GHz·μm^2^/particle	0.7 THz	Pattern material is gold layer with silver nanowires	[165]
Viruses	Hepatitis B virus DNA	SRR	Droplet deposition	LOD 127 IU/mL	0.95 THz	/	[129]
	Viruses HSV, HIV-I, and M13	L-shaped	Droplet deposition	1012 GHz/RIU	4.5 THz	Pattern material is InAs; polyamide film in the middle and gold substrate at bottom	[166]
Eukaryotes	Trypanosomes	Asymmetric double-split-ring resonator	Specific aptamer binding	/	/	/	[167]

## 4. Summary

We have reviewed the interdisciplinary research at the intersection of THz technology and microorganisms, encompassing two primary areas: the investigation of the effects of THz radiation on microorganisms and the development of THz-based techniques for microorganism detection. These studies have revealed unique biological effects and demonstrated promising applications, highlighting the potential of THz technology as a valuable tool for advancing microbial research and detection.

THz radiation has demonstrated different non-thermal effects compared to other stimuli. However, this area is still in its early stage and lacks comprehensive and systematic research. Some preliminary findings can be observed. For example, the response of microorganisms to THz radiation depends on the radiation parameters, including the center frequency, exposure time and power density. THz waves can interact with molecular processes such as gene transcription and protein translation, leading to changes in cellular activities. Compared with animal tissues, microorganisms possess unique characteristics such as ultrathin dimensions, autonomous growth, and rapid proliferation. These features can substantially amplify the effects of terahertz radiation, making microorganisms an ideal platform for the investigation of THz radiation effects.

Most literature shows positive cellular response (increasing DNA and protein expression) with respect to THz radiation. Future studies may utilize this characteristic to activate microbial cellular functions to increase the rate of reproduction or microbial products. It should also be noted that some studies suggested that THz radiation does not affect bacterial viability [75] or their metabolic activities [76], while exposure to approximately 6 J of total THz wave energy could even damage bacterial cells and cause cell death [77]. These effects should also be studied and considered in practical applications. While groundbreaking discoveries in this area might still be on the horizon, the potential of THz technology in microbiology remains promising and could reveal unforeseen insights and applications.

THz waves can also serve as tools to characterize, sense or identify microorganisms or their cellular components. THz measurements of living microorganisms have been mainly focused on bacteria *E. coli* and *Bacillus* spores, fungi and microalgae. Other types of microorganisms, such as *Mycobacterium tuberculosis* [158,159] and *S. aureus* [139,140,142,157] and *P. aeruginosa* [100,160,168] and protozoans of the genus *Trypanosoma* [167], were also investigated by different groups. For virus detection, coronavirus has received the most attention, while other infectious viruses have also been investigated either theoretically or experimentally, including avian influenza viruses (e.g., H1N1, H5N2 and H9N2) [136,137,148,149,150,151,162], bacteriophages [163,164,165], HIV [166] and hepatitis B virus [129].

THz spectroscopy reveals certain degrees of dielectric difference between different microorganisms, although the contrast is moderate. It is usually necessary to amplify these differences using field-enhancing techniques, typically MMs. This technique outperforms some commonly used biological methods by its relatively low cost, high sensitivity and rapid detection. In the spatial domain, THz s-SNOM can retrieve morphological and local dielectric properties from a single image scan, providing rich information for single-bacterium identification. However, the THz specificity against different microorganism species is still weak due to the lack of fingerprint absorptions in the THz range. THz thermal spectroscopy extracts more unique features from the temperature domain [145]. Although some works claimed that they have observed characteristic features, we highlight that these results are not supported by condensed matter theory and are more likely caused by errors or cavity oscillations. Therefore, developing binding techniques and combining them with THz sensors are important steps in the future to improve the target specificity, promoting the application of THz microorganism detection.

## 5. Challenges and Outlooks

### 5.1. Intense Water Absorption

**Figure 12:**
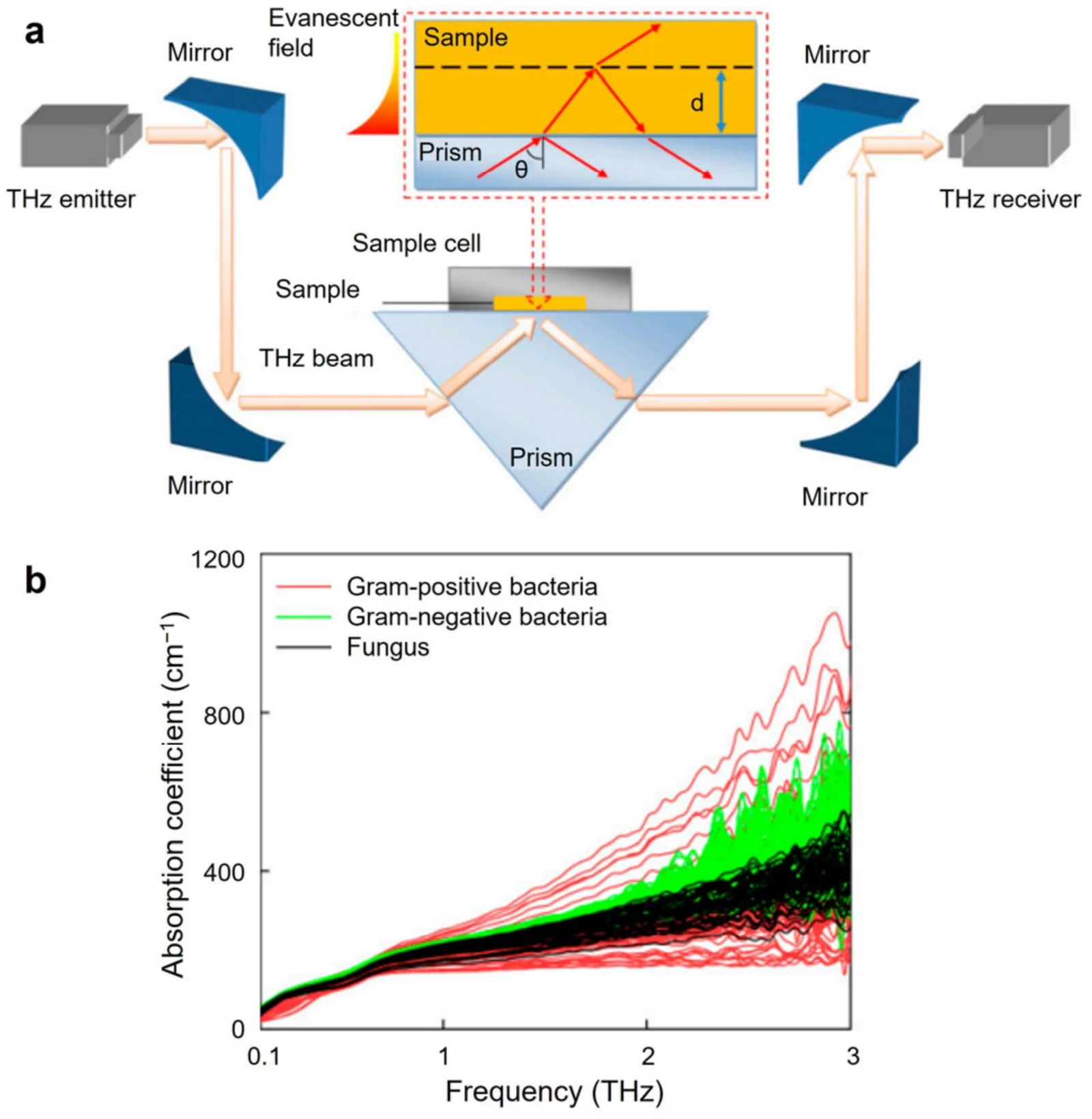
(**a**) Schematic illustration of the THz-ATR spectrometer with a sample cell made of Si. Inset: Diagram of the ‘prism–sample’ model. (**b**) THz absorption spectra of eight Gram-positive bacterial strains (red), two Gram-negative bacterial strains (green), and three fungi (black). Reproduced with permission from Yu et al., Biosensors 12, 378 (2022) [170]. Copyright 2022 the authors, licensed under a Creative Commons Attribution (CC BY) license.

The strong absorption of water limits the available SNR and bandwidth in THz transmission measurements. To minimize the influence, dehydration has been frequently applied. However, biomolecules and living things are only viable in an aqueous environment. It is important to use configurations more adaptive to hydrated biomaterials, such as defined-height sample chambers. The group from the Third Military Medical University of China did pioneering work on imaging living bacterial colonies with such designs [100,168]. Optical dielectric constants were measured for several common pathogenic bacterial species using a thickness-controllable sample chamber. They found that bacteria of different species or with different physiological states had varying hydration levels, and the small differences in water content led to distinguishable THz signals, which can be utilized for bacterial identification. Reflection configuration is another strategy to address the absorption issue [169]. In particular, attenuated total internal reflection (ATR) provides the best sensitivity for absorbing materials [170]. Yu et al. applied the ATR technique (Figure 12a) for clinical samples from sputum, blood, urine and feces and further used machine learning methods to analyze the spectrum. Bacterial stains isolated from different sample types and patient sources, or samples mixed with various biological components, are highly heterogeneous. The results show that thirteen standard microorganisms could be rapidly recognized and accurately classified into three groups of Gram-positive bacteria, Gram-negative bacteria, and fungi, owing to their different absorption coefficients (Figure 12b). The total diagnostic accuracy reached 80.77%.

**Figure 13:**
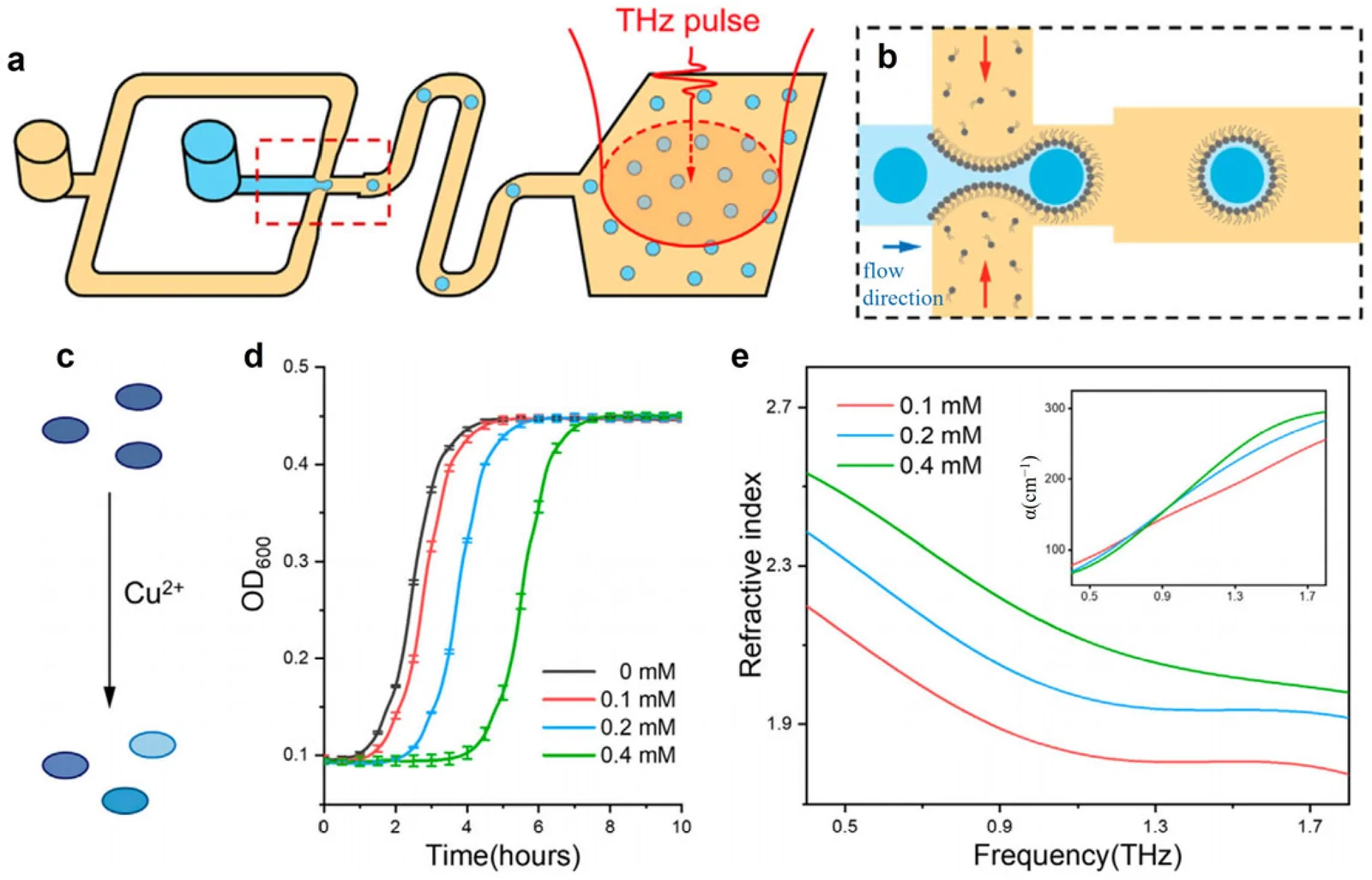
The integration of droplet microfluidics for automated and high-through performance. (**a**) Schematic of the microfluidic device that can generate droplet sample for THz-TDS measurement. An aqueous phase containing cells passes through a flow-focusing junction where it meets an oil phase containing lipids, leading to the generation of droplets. Red arrows show the position of a cell before and after encapsulation. Droplets are collected in a quartz chamber which serves as the THz measurement cuvette. (**b**) The schematic of the droplet generation process. (**c**) Schematic showing Cu^2+^ ion treatment induces distinct activity states of bacterial cells. (**d**) The growth curve of *E. coli* under 0, 0.1, 0.2 and 0.4 mM of Cu^2+^ treatment, respectively. (**e**) Refractive index and absorption coefficient spectra (inset) of *E. coli* cells. All bacterial samples were treated with Cu^2+^ and corresponded to the same cell numbers for THz measurement. Reproduced with permission from Zhang et al., Front. Bioeng. Biotechnol. 10, 1105249 (2023) [171]. Copyright 2023 the authors, licensed under a Creative Commons Attribution (CC BY) license.

Microfluidic devices work in a way similar to the defined-height chamber to balance the water absorption and sensitivity [144,161]. They offer another advantage of flexible input–output control that supports in vitro stimulus applications. Many soft polymers exhibit low loss in the THz range, such as cyclic olefin copolymer (COC) and polydimethylsiloxane (PDMS). They are widely applied in fabricating THz microfluidic devices. Zhang et al. [171] developed a microfluidic cellular encapsulation device to measure the refractive index of living bacteria in near-physiological environments (Figure 13). The viable cells were encapsulated in aqueous droplets at a moderate thickness. The physiological states of bacteria changed with external stimulus of copper ions (Cu^2+^), and they found the refractive index of bacterial droplets increased with increasing Cu^2+^ concentration. The use of a microfluidic device enables differentiating physiological states of bacteria under stress conditions. However, sensitive techniques for highly hydrated samples are still limited, especially for field-enhancing sensors. The resonant nature of most sensors suffers from the strong damping effect caused by the absorptive environment, significantly reducing their Q-factors. Reflection-type sensors with a shorter field-confining area may address these issues [172].

Although the above strategies enable measuring of living microorganisms in hydrated environments, the measurement sensitivity is often limited. In transmission measurements, a trade-off exists between the optical interaction path length and attenuation: thinner samples reduce absorption losses but also produce weaker contrast because of the reduced light–sample interaction. In reflection measurements, the strong absorption of water, manifested by its large imaginary refractive index, likewise limits the sensitivity. By contrast, dehydration can substantially improve measurement sensitivity by reducing water absorption, but at the expense of cell viability.

### 5.2. Poor Spatial Resolution

**Figure 14:**
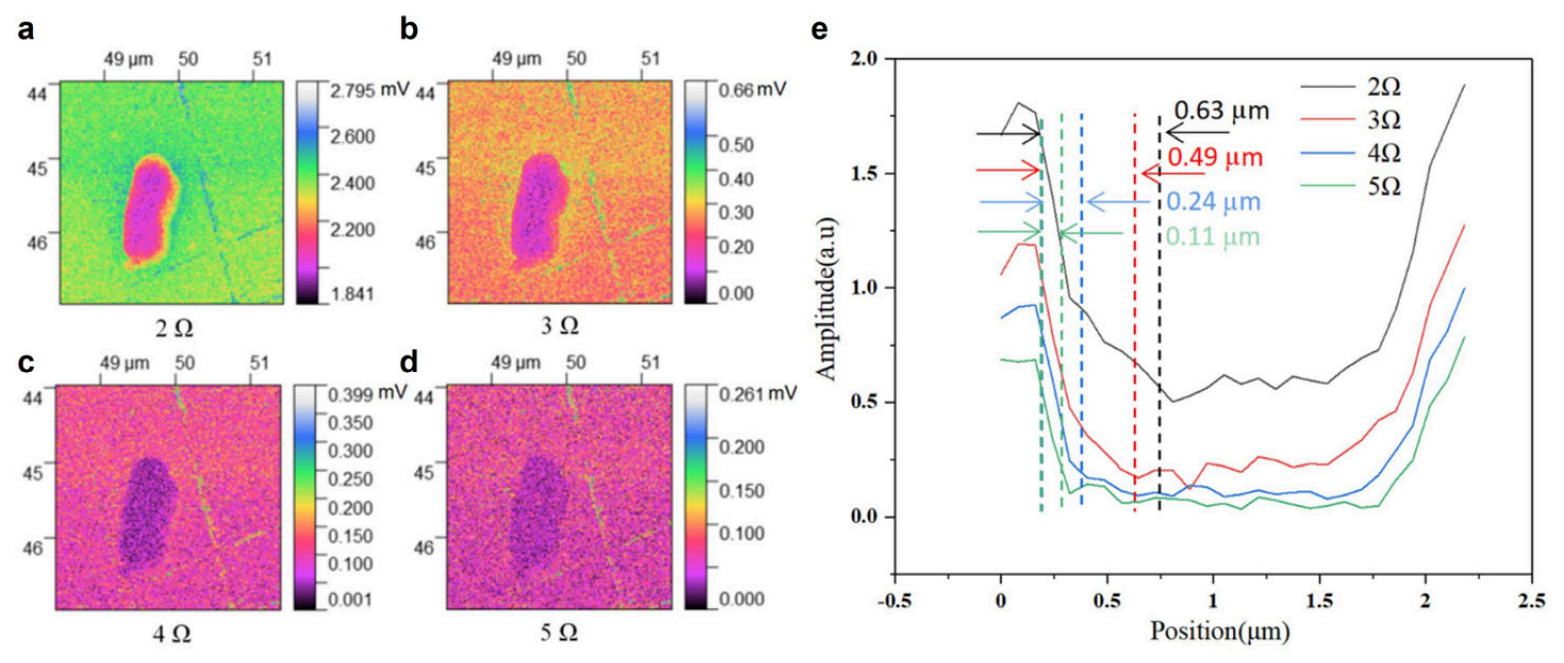
THz s-SNOM images of *E. coli*, demodulated at different harmonic frequencies of (**a**) 2 Ω, (**b**) 3 Ω, (**c**) 4 Ω and (**d**) 5 Ω, respectively. (**e**) Spatial resolution of THz near-field images of *E. coli*, analyzed by the signal amplitude variation across the bacteria, for various harmonic orders of signal demodulation. Spatial resolution is estimated based on the spatial spread of the point where the signal is 90% of its maximum to the point where the signal is 10% of its maximum. Reproduced with permission from Wang et al., Front. Microbiol. 14, 1195448 (2023) [119]. Copyright 2023 the authors, licensed under a Creative Commons Attribution (CC BY) license.

THz imaging suffers from poor spatial resolution due to the diffraction limit, especially at low frequencies. The far-field resolution is over ten times higher than that of a single cell, making it only applicable for bulk mixtures. The improvement factors of various far-field resolution-enhancing techniques are typically smaller than 10 [173,174,175], making them only applicable for large-scale samples. The detection of subtle changes inside microorganisms is nearly impossible, which hinders the sensitivity and specificity. Going beyond the diffraction limit requires near-field technologies. THz s-SNOM is an efficient approach to address this issue. The resolution of THz s-SNOM can reach as low as a few tens of nanometers, which is fundamentally set by the tip radius rather than the wavelength of illumination. The technique utilizes a nanoscale tip oscillating at a certain frequency to scatter the THz near fields and demodulate it from the far-field signal based on the lock-in detection principle. THz s-SNOM has been used to investigate bacterial single-cell and subcellular structures [116,117,118,119,120], as introduced in Section 3.2.1. Technically, the spatial resolution of THz s-SNOM is further affected by the harmonic order used to reconstruct the images. Higher orders lead to a better spatial resolution but a lower signal-to-noise ratio. Wang et al. [119] compared THz s-SNOM images of E. coli at harmonic frequencies of 2 Ω, 3 Ω, 4 Ω and 5 Ω, respectively (Figure 14a–d). Figure 14e compares the amplitude variation across the bacteria region, showing the improved resolution and reduced signal strength with the increased harmonic order. Therefore, increasing the dynamic range of THz systems is also important in improving the spatial resolution of THz near-field images.

### 5.3. Experimental Standards and Data Reproducibility

Considering that THz biophotonics is a relatively new research area in the last two to three decades, it is reasonable that standards have not been established for THz measurements. This is more difficult for biomedical applications because samples have varying types, forms and preparation methods. However, the lack of experimental standards makes it difficult to evaluate the data reliability and accuracy. Improper data processing results in errors, such as the spectral features claimed to have been observed in aqueous samples or mixtures of biological macromolecules. Another consequence is the low data reproducibility. Reported works are highly independent of each other, making data comparison nearly impossible. Particularly, studies investigating radiation effects used different sources, power, exposure time, target samples and evaluation methods. Establishing standards in experimental setups, sample preparation and data processing and encouraging more studies to reproduce and verify the existing works are important steps to improve the data quality in this field. Establishing universally applicable numerical standards for the irradiation parameters in THz biological studies is impractical, as many of these parameters, such as frequency, power density, peak electric-field strength, and exposure duration, are themselves experimental variables that determine the biological response. Nevertheless, to improve the transparency, reproducibility, and comparability of different studies, future work is strongly encouraged to provide a comprehensive description of the irradiation and experimental conditions, including the key electromagnetic, thermal, and biological parameters summarized in Table 4. Whenever possible, consistent units and reporting conventions should be adopted to facilitate quantitative comparisons across studies.

### 5.4. Outlooks

THz is a virgin frequency range with unique features and advantages in many interdisciplinary research areas. Microorganisms consist of a giant number of species that can adapt to a wide range of living environments, making them well-suited to the experimental conditions of THz measurements. Compared to animal tissues, the independent survival characteristics of microorganisms, along with their rich metabolic activity, are especially adaptive to the limited penetration depth of THz waves in hydrated samples. The interdisciplinary area between THz photonics and microorganisms has many interesting unexplored phenomena, theories and applications to be revealed. Although many challenges are yet to be addressed, this research area attracts growing amounts of attention, with new findings and technologies being reported in recent years. With ongoing research, terahertz technology is expected to become a novel research tool or detection technique in the field of microbiology, promoting the advancement of both areas.

## Figures and Tables

**Table 1:** Major technologies applied for microorganism studies.

Major Category	Representative Techniques	Main Information Obtained
Microscopy and imaging	Bright-field microscopy, phase-contrast microscopy, fluorescence microscopy, confocal microscopy, electron microscopy	Morphology, size, structure, localization, viability
Culture-based and phenotypic methods	Culture, colony morphology, selective/differential media, Gram staining, biochemical tests, metabolic assays	Growth characteristics, morphology, metabolic/physiological properties
Immunological methods	Enzyme-linked immunosorbent assay (ELISA), immunofluorescence, lateral-flow assays, immunomagnetic separation	Specific antigens/cellular components
Nucleic-acid-based methods	Polymerase chain reaction (PCR), reverse transcription PCR (RT-PCR), quantitative PCR (qPCR), rRNA sequencing	Genetic identity, abundance, genetic composition
Spectroscopic and chemical analysis	Fourier transform infrared (FTIR), Raman spectroscopy, fluorescence spectroscopy, nuclear magnetic resonance (NMR)	Molecular composition, biochemical fingerprints, chemical states
Cytometric and single-cell methods	Flow cytometry, imaging flow cytometry, cell sorting	Cell abundance, size, morphology, viability, physiological heterogeneity
Omics and systems-level approaches	Metagenomics, metatranscriptomics, metaproteomics, metabolomics	Community composition, gene expression, proteins, metabolites

**Table 4:** Recommended parameter list for studies on THz radiation effect.

Parameters	Units/Options
THz source type	—
Frequency	THz
Bandwidth	GHz/THz
Operation mode	Pulsed/CW
Pulse duration	ps/ns/μs
Repetition rate	Hz/kHz/MHz
Spot size	mm^2^
Average power density	mW/cm^2^
Peak electric-field strength	kV/cm
Exposure duration	min/h
Temperature and variation	°C
Cell concentration	CFU/mL
Cell growth phase	Exponential or stationary
Sample volume	mm × mm × mm

## Data Availability

No new data were created or analyzed in this study. Data sharing is not applicable to this article.

## Abbreviations

The following abbreviations are used in this manuscript:

AFM	atomic force microscope
ATR	attenuated total reflection
*B. subtilis*	*Bacillus subtilis*
CFU	colony-forming unit
COVID-19	coronavirus disease 2019
CW	continuous wave
*E. coli*	*Escherichia coli*
ELISA	enzyme-linked immunosorbent assay
EPSs	extracellular polymeric substances
FEL	free electron laser
FTIR	Fourier-transform infrared
FWHM	full width at half maximum
*G. icigianus*	*Geobacillus icigianus*
GMNM	gold magnetic nanoparticle-mediated
GFP	green fluorescent protein
HBV	hepatitis B virus
LOD	limit of detection
MMs	metamaterials
QCL	quantum cascade laser
Q-factor	quality factor
qPCR	quantitative polymerase chain reaction
RIU	unit refractive index change
RCA	rolling circle amplification
*S. aureus*	*Staphylococcus aureus*
*S. epidermidis*	*Staphylococcus epidermidis*
SARS-CoV-2	severe acute respiratory syndrome coronavirus 2
SCENIHR	the Scientific Committee on Emerging and Newly Identified Health Risks
SPPs	surface plasmon polaritons
SRR	split-ring resonator
s-SNOM	scattering-type scanning near-field optical microscope
TDS	time-domain spectroscopy
THz	terahertz
UV	ultraviolet
